# Mechanical equipment fault diagnosis method based on improved deep residual shrinkage network

**DOI:** 10.1371/journal.pone.0307672

**Published:** 2024-10-28

**Authors:** Shaoming Qiu, Liangyu Liu, Yan Wang, Xinchen Huang, Bicong E., Jingfeng Ye

**Affiliations:** School of Information Engineering, Dalian University, Dalian, Liaoning, China; University of Southern California, UNITED STATES OF AMERICA

## Abstract

Fault diagnosis of mechanical equipment can effectively reduce property losses and casualties. Bearing vibration signals, as one of the effective sources of diagnostic information, are often overwhelmed by substantial environmental noise. To address this issue, we present a fault diagnosis method, CCSDRSN, which exhibits strong noise resistance. This method enhances the soft threshold function in the traditional deep residual shrinkage network, allowing it to extract useful information from the fault signal to the maximum extent, thus significantly improving diagnostic accuracy. Additionally, we have developed a novel activation function that can nonlinearly transform the time frequency map across multiple dimensions and the entire region. In pursuit of network optimization and parameter reduction, we have strategically incorporated depthwise separable convolutions, effectively replacing conventional convolutional layers. This architectural innovation streamlines the network. By verifying the effectiveness of the proposed method using Case Western Reserve University datasets, the results demonstrate that the proposed method not only possesses strong noise resistance in high noise environments but also achieves high diagnostic accuracy and good generalization performance under different load conditions.

## Introduction

Mechanical equipment is a cornerstone of modern industrial production. However, these vital machines often operate in harsh conditions, leading to frequent malfunctions that can result in significant economic losses and, in the worst cases, even fatalities. Consequently, the detection and diagnosis of faults in mechanical equipment is imperative.

As industrial society rapidly evolves, machines are becoming more intelligent and sophisticated, rendering traditional model based diagnostic methods increasingly impractical. The operating environments of mechanical equipment in sectors such as industrial production, aerospace, and high speed rail are complex and dynamic, and early fault signals are often faint and easily drowned out by noise. As a result, methods that rely solely on signal processing struggle to achieve high diagnostic accuracy and are heavily dependent on the expertise of specialists.

In recent years, the substantial enhancement of computer performance and the advent of big data have propelled deep learning methods to the forefront of mechanical equipment fault diagnosis. These advanced techniques can automatically extract valuable information from raw vibration signals and identify early fault indicators, despite the presence of weak and obscured effective signals, thereby significantly improving diagnostic precision.

A multitude of advanced deep learning techniques, including Long Short Term Memory (LSTM) networks, Convolutional Neural Networks (CNNs), Deep Belief Networks (DBNs), Stacked Autoencoders, Deep Residual Shrinkage Networks, and Deep Transfer Learning, have been adeptly employed by experts in the domain of fault diagnosis to detect anomalies across a broad spectrum of mechanical production equipment. For instance, Chen et al. [1] used convolutional neural networks with different kernel sizes to adaptively extract features of different frequency signals, input the learned features into the long short term memory network for fault type identification, and perform downsampling before data input, greatly reducing the number of training parameters. Chang et al. [2] used variational mode decomposition to process the original voltage signal, then used continuous wavelet transform to convert the signal into a time frequency diagram, and finally used a clustering algorithm to classify these time frequency diagrams. Zhang et al. [3] proposed a rotating machinery fault type identification method based on recursive neural networks to address the shortcoming of convolutional neural networks that ignore the information of time series signals. This method focuses on learning representative features from the time information of the data, using a multi-layer perceptron (MLP) to implement fault identification. Zhao et al. [4] combined wavelet packet distortion and convolutional neural network, using distorted wavelet packet coefficients to increase fault samples, and finally inputting them into the classification model of the convolutional neural network. Chen et al. [5] proposed an improved fault diagnosis method based on a continuous wavelet transform local binary convolutional neural network model, which automatically diagnoses fault categories of rotating machinery by capturing the fault characteristics of vibration signals. This binary convolution has a faster training speed than ordinary convolution. Zhao et al. [6] proposed a vibration amplitude spectrum imaging feature extraction method based on continuous wavelets, which can extract two dimensional image features and eliminate manual features that affect low signal-to-noise ratio conditions. Chen et al. [7] proposed a method based on convolutional neural network and discrete wavelet to identify the fault type of planetary gearbox, addressing the highly nonstationary and nonlinear problem of planetary gearbox vibration signals.

The data of equipment operation status can not only be used for fault diagnosis but also for the remaining useful life (RUL) prediction of various components of mechanical equipment. For example, Ni et al. [8] inferred the degradation progression by developing a novel health indicator (HI) and employed the gated recurrent unit network to predict the RUL of the bearing system. Feng et al. [9] developed a digital twin-driven intelligent health management method to monitor and assess gear surface degradation progression. This method can effectively reveal the gear wear propagation characteristics and predict the RUL accurately. Wu et al. [10] proposed a temporal multiresolution hypergraph attention network (T-MHGAT), where a hypergraph attention network (HGAT) is designed to mine the high order relationships between signal samples on the hypergraph data.

Zhao et al. [11] delve into the prevalent unsupervised deep transfer learning settings and algorithms, establishing a novel taxonomy for intelligent fault diagnosis based on these methods. Chen et al. [12] provide a comprehensive review of the advancements in deep transfer learning for bearing fault diagnosis since 2016, categorizing the literature by target domain data properties and offering an in depth analysis of the prevalent deep transfer learning methodologies. The potential of deep transfer learning in the realm of intelligent fault diagnosis continues to be explored by researchers. Ding et al. [13] introduce an innovative framework for deep imbalanced domain adaptation (DIDA) in bearing fault diagnosis, tackling the complex scenario where feature and label shifts occur simultaneously under varying operating conditions, with a focus on overcoming class imbalanced label shifts. Li et al. [14] address the challenges of extracting fine grained fault features and enhancing model generalization for unseen few shot faults in few shot fine grained fault diagnosis tasks by proposing a novel attention based deep meta transfer learning (ADMTL) approach.

Traditional deep learning methodologies often encounter issues such as gradient vanishing and exploding, particularly as network layers become more numerous. The Deep Residual Network (ResNet), introduced by He et al. [15], represents a groundbreaking evolution in the field of convolutional neural networks. By incorporating an identity path for parameter optimization, ResNet enables the residual network to not only perform layer by layer inversion but also to propagate data through a constant path, returning to the initial layer. Liang et al. [16] have successfully applied wavelet transformation in conjunction with an enhanced residual network (ResNet) to detect faults in rolling bearings within large scale mechanical equipment. Ma et al. [17] have proposed a fault diagnosis approach that leverages time frequency analysis and the deep residual network, capable of delivering effective diagnostics through direct spectral analysis without the need for specific dynamic knowledge. Ni et al. [18] proposed a novel Physics Informed Residual Network (PIResNet) for learning the underlying physics that is embedded in both training and testing data, thus providing a physically consistent solution for imperfect data. Zhao et al. [19] have developed an innovative activation function that enables each input signal to undergo a set of unique nonlinear transformations. This function is then integrated into the deep residual network, demonstrating the method’s superior performance in practice.

However, most existing network models in fault diagnosis heavily rely on ideal vibration signals. In actual production environments, the original vibration signals of mechanical equipment often contain a large amount of noise, which can drown out some fault related features. In such cases, the diagnostic accuracy of most deep learning network models will decrease.

Therefore, Zhao et al. [20] utilized the residual network combined with the idea of attention to integrate the soft threshold from traditional signal processing into the deep architecture, developing a Deep Residual Shrinkage Network (DRSN) capable of adaptively reducing vibration signals. Numerous experimental results demonstrate that the fault diagnosis accuracy of the DRSN under high noise and complex working conditions surpasses that of stacked autoencoders, convolutional neural networks, and residual networks. Currently, DRSNs have garnered increasing attention and study. For instance, Yang et al. [21] configured the first layer of the one dimensional deep residual shrinkage network as a wide convolution layer for fault diagnosis on high noise signals. Although the receptive field is expanded, the ability to suppress noise is still lacking and the generalization ability of the model is poor. Hu et al. [22] proposed a variable soft threshold to eliminate constant deviation and applied it to transformer fault identification, demonstrating DRSN’s scalability in power applications and fault prevention. However, the overall delinearization capability of the model is insufficient and it cannot perform adaptive nonlinear transformation on the signal. Salimy et al. [23] employed DRSN for classification and denoising when using electromagnetic interference technology to capture fault signals of high voltage power equipment.

Pei et al. [24] proposed a novel method to enhance the Deep Residual Shrinkage Network (DRSN) by utilizing the feature and noise energy ratio as the evaluation index for bearing performance degradation. This approach addresses the challenge of accurately identifying the degradation state of rolling bearings. Tong et al. [25] further refined the soft threshold function and introduced the Improved Pseudo Soft Threshold Function (IPSTF) to mitigate signal distortion issues caused by the original soft threshold function. However, it is easy to suppress effective information. Zhang et al. [26–29] enhanced the soft threshold function within the original DRSN framework by proposing a soft threshold function with adaptive slope, which more effectively eliminates noise in the signal. This threshold function easily causes the problem of insufficient feature extraction capability of the input signal due to excessive contraction of the threshold.

Most of the above models are still not capable of processing noise, and the useful information in the fault signal is not effectively utilized. In order to solve these problems and To fully leverage the superior characteristics of the Deep Residual Shrinkage Network (DRSN) in processing high noise signals, this article aims to further enhance the DRSN model.

Most of the current mainstream methods try to extract useful information from the signal, but few have the ability to suppress noise, or the ability to suppress noise is insufficient.the inherent soft threshold function within the DRSN, although effective in noise reduction, may inadvertently lead to the loss of valuable signal information, thus diminishing the network’s feature learning capacity. To address this issue, this study introduces an enhanced soft threshold function designed to preserve critical signal details while mitigating noise interference. Furthermore, this research innovates by crafting a novel activation function, inspired by GPReLU (Global Parametric Rectified Linear Unit), to enrich the network’s nonlinear transformation capabilities.

This article integrates the Continuous Wavelet Transform (CWT), the attention mechanism of the convolution module (Convolutional Block Attention Module, CBAM), and the separable convolution (Separable Convolution, SC) to develop a fault diagnosis model with strong noise resistance, namely CCSDRSN. This network achieves improved fault diagnosis accuracy under high noise conditions.

The main contributions of this article are summarized as follows:

1. A new soft threshold function is developed to enhance its ability to extract fault features and prevent excessive shrinkage of the threshold.
2. Developing a new activation function and integrating it into the Deep Residual Shrinkage Network (DRSN) enables the network to capture useful information in the time frequency diagram from both spatial and channel directions, thereby enhancing fault diagnosis accuracy.
3. To reduce the number of parameters in the residual shrinkage module, shorten training time, and achieve separation of convolution channels and convolution areas, ordinary convolution in the residual shrinkage module is replaced with depthwise separable convolution.

The remainder of this article is structured as follows: Section 2 provides a brief overview of the classic DRSN model and analyzes the existing issues within the model. Section 3 outlines the data preprocessing method used in this study, details the improved DRSN structure, and describes the enhancements made to an activation function. A comprehensive summary of the fault diagnosis model developed in this article is presented. In Section 4, experimental comparisons are conducted. Finally, Section 5 summarizes the main contributions of this article.

## DRSN model

The Deep Residual Shrinkage Network (DRSN) is an improvement over ResNet. It introduces the soft threshold from traditional signal processing as a shrinkage function to construct a Residual Shrinkage Building Unit (RSBU). Multiple RSBUs can be utilized within the network architecture. RSBU effectively suppresses noise that is not related to the current pattern recognition information, thereby achieving noise reduction.

**Fig 1:**
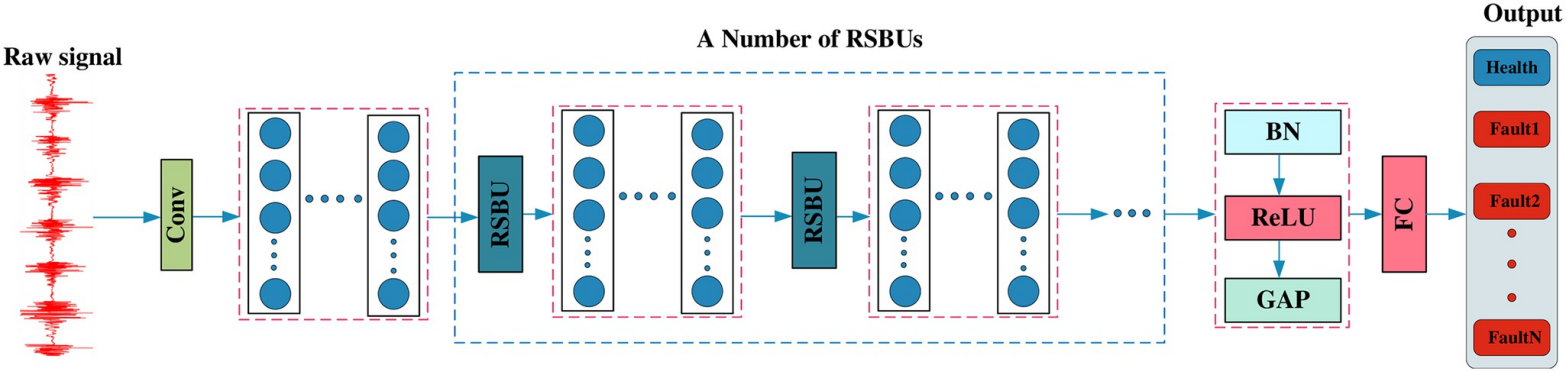
The composition of the DRSN.

As can be seen from Fig 1, the component units of DRSN include the convolution layer, residual shrinkage module, batch normalization (Batch Normalization, BN), activation function (Rectified Linear Unit, ReLU), Global Average Poling (GAP).

The activation function is mainly used for nonlinear transformation in neural networks, and ReLU can effectively prevent the gradient from disappearing. The ReLU activation function can be expressed as Eq (1):

$$\begin{array}{c} y=max(0,x) \end{array}$$ (1)

Where *x* is the input feature and *y* is the output feature, *max*(0, *x*) represents taking the larger value between the input value and zero.

**Fig 2:**
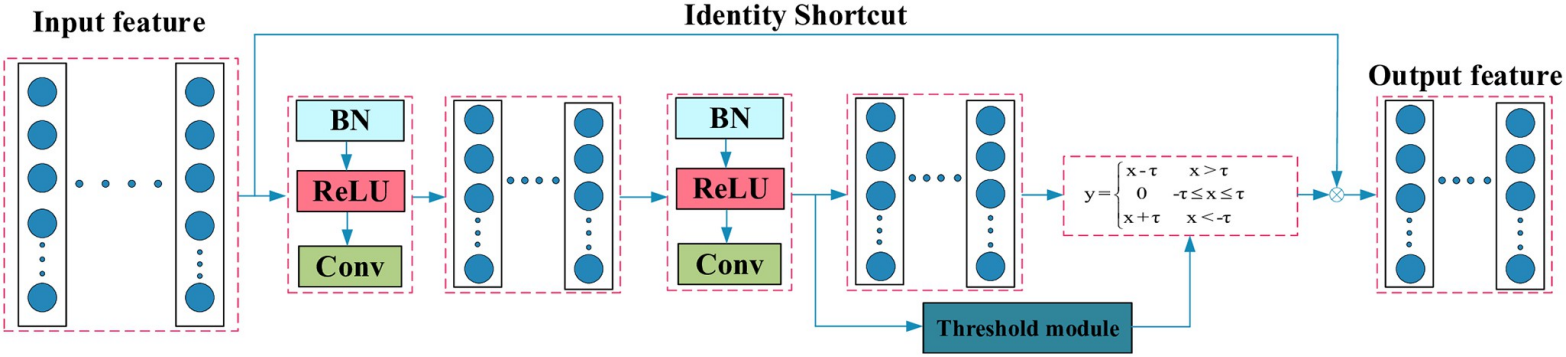
The composition of the RSBU.

The residual shrinkage module is depicted in Fig 2. As illustrated in the figure, the soft threshold function is capable of acquiring different thresholds based on varying input features. Ultimately, the soft thresholded features are added to the input features to extract filtered noise characteristics.

**Fig 3:**
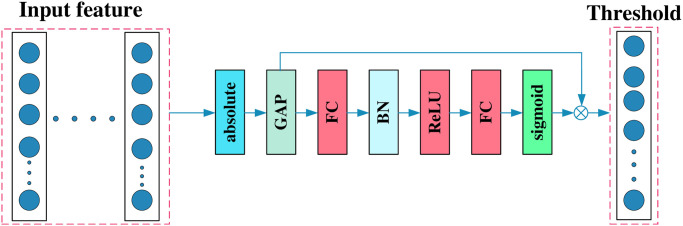
The composition of the threshold module.

The detailed process of threshold acquisition is shown in Fig 3. According to the characteristics of the threshold, operations such as Absolute, BN, ReLU, and Sigmoid are used to norm the threshold.

In addition to the soft threshold function, the Deep Residual Shrinkage Network (DRSN) shares similarities with the residual network, encompassing operations such as convolution modules, batch normalization, global mean processing, and fully connected layers. However, the soft threshold function serves as the core step of the noise reduction method in this network. The soft threshold function in DRSN is represented as Eq (2).

$$\begin{array}{c} y=\{\begin{array}{cc} x-\tau & \;\;x>\tau \\ 0 & \;-\tau ⩽x⩽\tau \\ x+\tau & \;\;x<\tau \end{array} \end{array}$$ (2)

**Fig 4:**
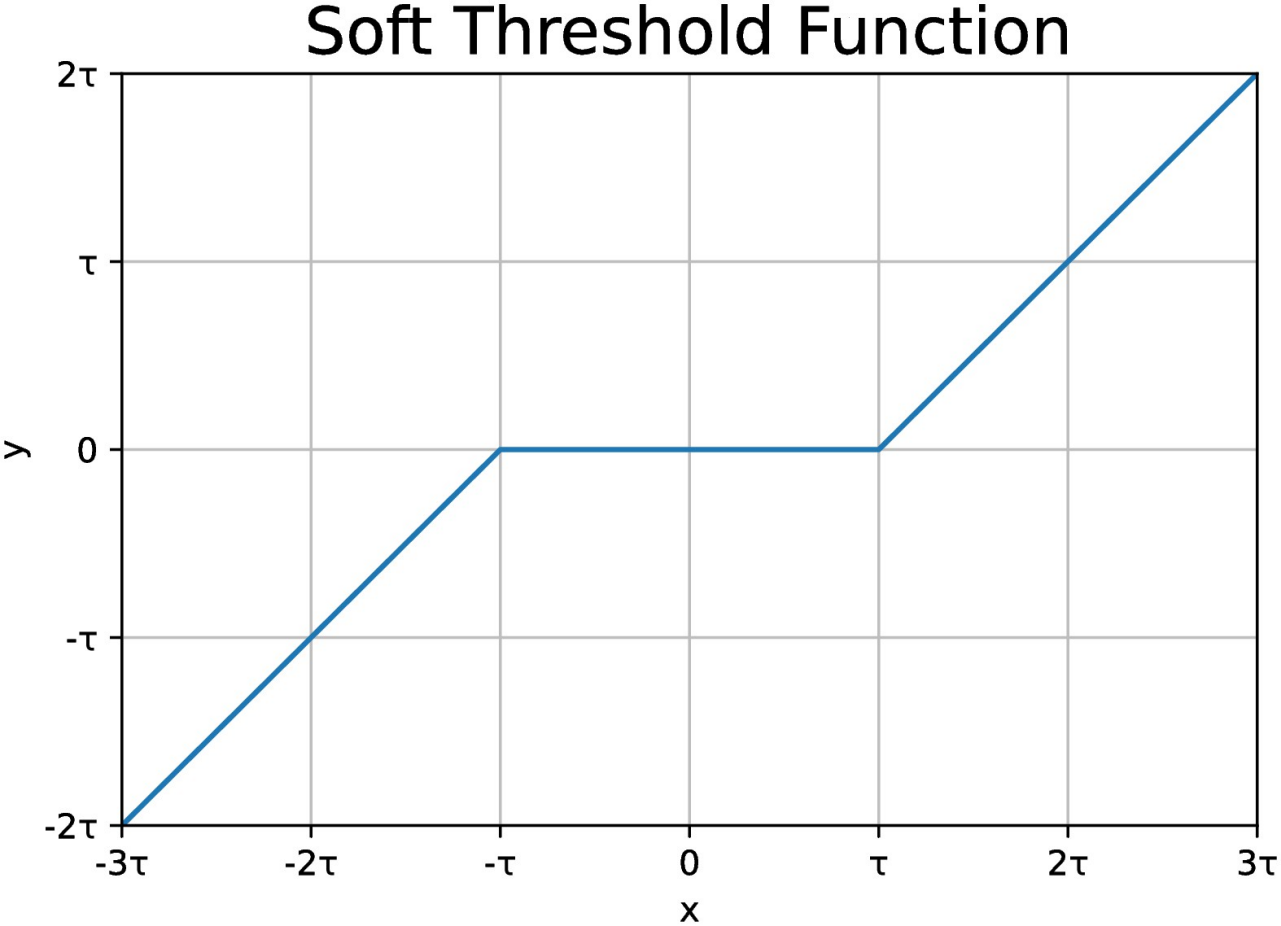
Soft thresholding function.

Among them, *x* represents the input feature, *y* represents the output feature, and *τ* is the threshold. The soft threshold function can set the features close to zero in the ReLU activation function to zero, so that the useful information in the negative feature area is retained. The image of the soft threshold function is shown in Fig 4.

The soft threshold function, while effective in eliminating noise, tends to suppress useful information along with the noise. Additionally, it faces the challenge of insufficient fault feature extraction due to excessive threshold shrinkage.

When diagnosing faults based on one dimensional time series signal inputs, it’s crucial to address the issue of insufficient time domain information in the original signal. Furthermore, deep stacking of RSBU can lead to redundant calculation parameters and prolonged training time.

To mitigate these challenges, this article initially introduces continuous wavelet transform for signal preprocessing. Subsequently, it improves both the soft threshold function and the residual shrinkage module.

## The CCSDRSN fault diagnosis model

### Continuous wavelet transform

The Continuous Wavelet Transform (CWT), an advancement of the Fourier Transform, serves as a potent tool for extracting time frequency information from signals. During the process of decomposition and reconstruction of the input signal, a “mother wavelet” with rapid attenuation or finite length is utilized. This mother wavelet adapts to the input signal via scale adjustment and wavelet translation. Unlike the Fourier Transform, which struggles to capture temporal features within signals, the mother wavelet function employed in CWT addresses this limitation. CWT surpasses the Short Time Fourier Transform (STFT) in handling abrupt changes and nonstationary signals. Its primary advantage lies in its capability to extract signal features from both temporal and frequency domains simultaneously. CWT provides superior frequency resolution for low frequency signals and enhanced time resolution for high frequency signals.

The formula of continuous wavelet transform is as follows:

$$\begin{array}{c} CWT\left(a,b\right)=\left\langle f,\psi_{a,b}\right\rangle =\frac{1}{\sqrt{a}}\int_{-\infty }^{+\infty }f\left(t\right)\cdot \psi^{*}(\frac{t-b}{a})dt \end{array}$$ (3)

Where *ψ*_*a*,*b*_ is the mother wavelet function, $\psi^{*}(\frac{t-b}{a})$ is the conjugate operation of $\psi (\frac{t-b}{a})$, *f*(*t*) is the original time domain signal, 〈*f*, *ψ*_*a*,*b*_〉 is a series of transformation coefficients obtained after the inner product operation of any square integrable function *f*(*t*) and the mother wavelet function *ψ*_*a*,*b*_. Through this coefficient, a time frequency signal with good time and frequency domain localization can be constructed.

By scaling and delaying the mother wavelet function *ψ*_*a*,*b*_, a series of wavelet basis functions can be obtained. The transformation process can be expressed as:

$$\begin{array}{c} \psi_{a,b}=\frac{1}{\sqrt{a}}\psi (\frac{t-b}{a}),\;a,b\in R,\;\;\;a>0 \end{array}$$ (4)

During the transformation process, the frequency is located through parameter *a*, and the time is located through parameter *b*. At the starting position of a segment of signal, compare the mother wavelet function and the signal to obtain the wavelet coefficient *WT*(*a*, *b*), which is 〈*f*, *ψ*_*a*,*b*_〉. Then move the wavelet function along the horizontal time axis, that is, change the parameter, and recalculate the wavelet coefficient until the signal cutoff position. Finally, the time frequency information of this section of signal is obtained, that is, the result of continuous wavelet transform *CWT*(*a*, *b*).

*CWT*(*a*, *b*) reflects the similarity between the original signal and the mother wavelet function through the inner product operation between the two, so the mother wavelet function should be similar to the fault pulse characteristics. The Morlet wavelet selected in this article has similar characteristics to pulse. The expression of Morlet wavelet is:

$$\begin{array}{c} \psi \left(t\right)=\pi^{-\frac{1}{4}}e^{-\frac{t^{2}}{2}}\;\text{cos}\left(w_{0}t\right) \end{array}$$ (5)

Among them, *t* is time and *w*_0_ is the center frequency of wavelet.

### Improved adaptive leaky thresholding

Building upon the findings in [27], due to the problem of the threshold deviation in the soft threshold function is large and useful information being compressed. this article has improved the soft threshold function and threshold interval. As shown in Eq (2), the original soft threshold interval is [−*τ*, *τ*]. In order to expand the extraction of effective information, the threshold interval is improved to [−*τ*^2^, *τ*^2^]. The improved soft threshold function expression is as follows:

$$y=\{\begin{array}{cc} x+\left(\alpha -1\right)\tau^{2} & \;\;x>\tau^{2}\\ \alpha \cdot x & \;-\tau^{2}⩽x⩽\tau^{2}\\ x+\left(1-\alpha \right)\tau^{2} & \;\;x<-\tau^{2} \end{array}$$ (6)

**Fig 5:**
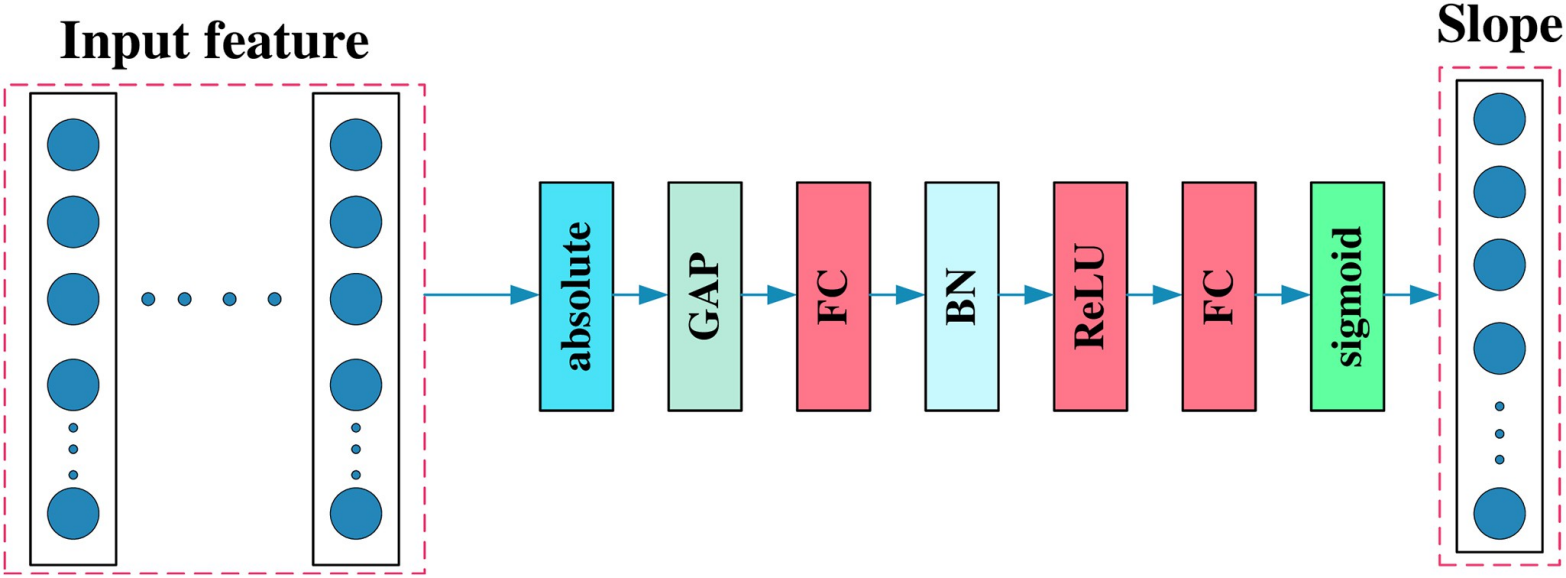
The composition of the slope module.

**Fig 6:**
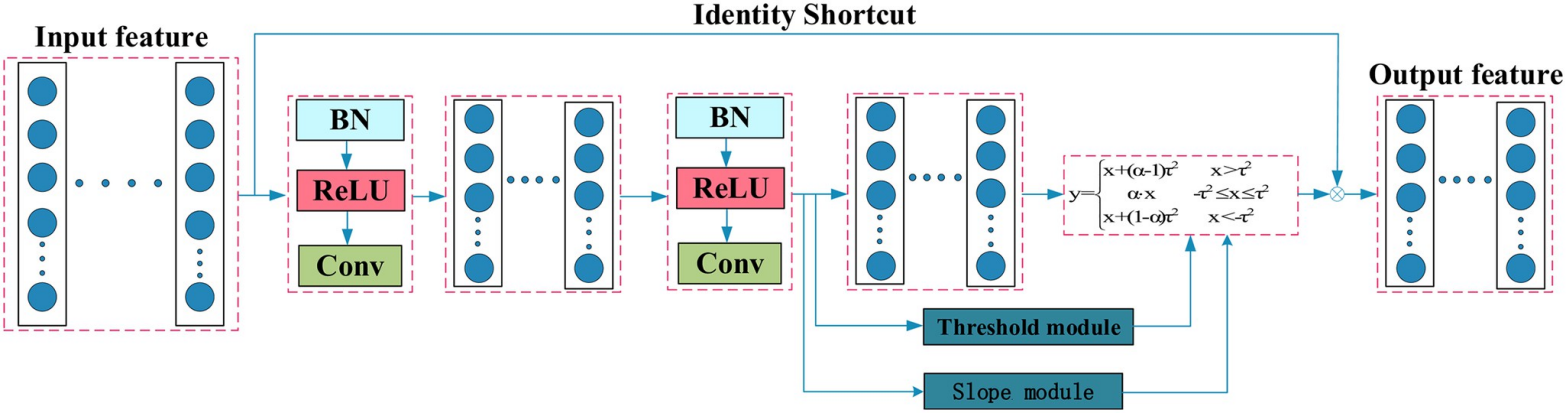
The composition of the improved RSBU.

Combine slope *α* with soft threshold *τ*, The slope control threshold area is used to extract features, and the process of obtaining the slope is shown in Fig 5. The RSBU module after changing the soft threshold is shown in Fig 6.

### Improved activation function-CGPReLU

Zhao et al. [19] in order to solve the problem that the ReLU activation function can only perform a fixed linear transformation on the input signal, an adaptive parameter rectified linear unit, namely APReLU (Adaptive Parameter ReLU, APReLU), was developed. It can be explained by the following expression:

y=max(x,0)+α·min(x,0) (7)

This activation function is improved from LReLU(Leaky ReLU). LReLU multiplies negative features by a small nonzero coefficient, such as 0.01, but does not force them to be zero. The expression of LReLU is as follows:

$$\begin{array}{c} y=\text{max}\left(x,0\right)+0.01\cdot \;\text{min}\;\left(x,0\right) \end{array}$$ (8)

Where *x* and *y* are input and output features respectively.

The Adaptive Parametric Rectified Linear Unit (APReLU) enables each input signal to undergo its own nonlinear transformation, thereby enhancing feature extraction capabilities under complex working conditions. In the expression provided, *α* represents a multiplicative coefficient obtained through training of the input signal, corresponding to the slope. However, APReLU solely performs nonlinear transformation on signals within the negative feature area, neglecting the positive feature area. To address this limitation, Zhang et al. [29] proposed an improvement known as the Global Parametric Rectified Linear Unit (GPReLU), characterized by the following expression:

$$\begin{array}{c} y=\beta \cdot \;\text{max}\;\left(x,0\right)+\alpha \cdot \;\text{min}\;\left(x,0\right) \end{array}$$ (9)

**Fig 7:**
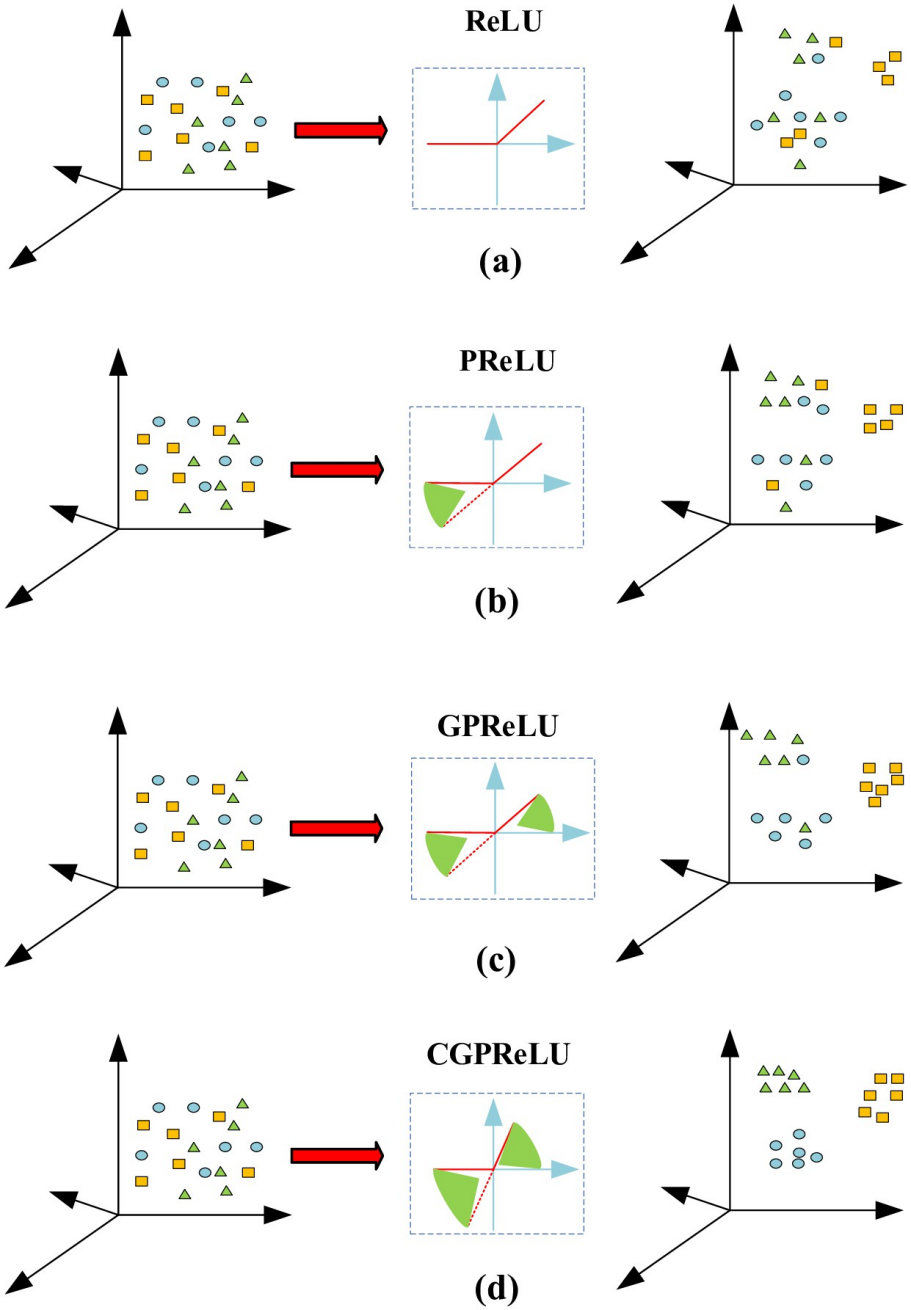
Schematic diagram of nonlinear transformation of different activation functions.

**Fig 8:**
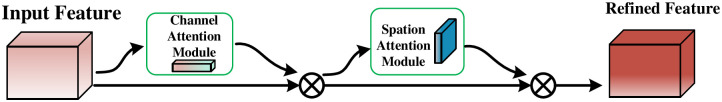
The overview of CBAM.

**Fig 9:**
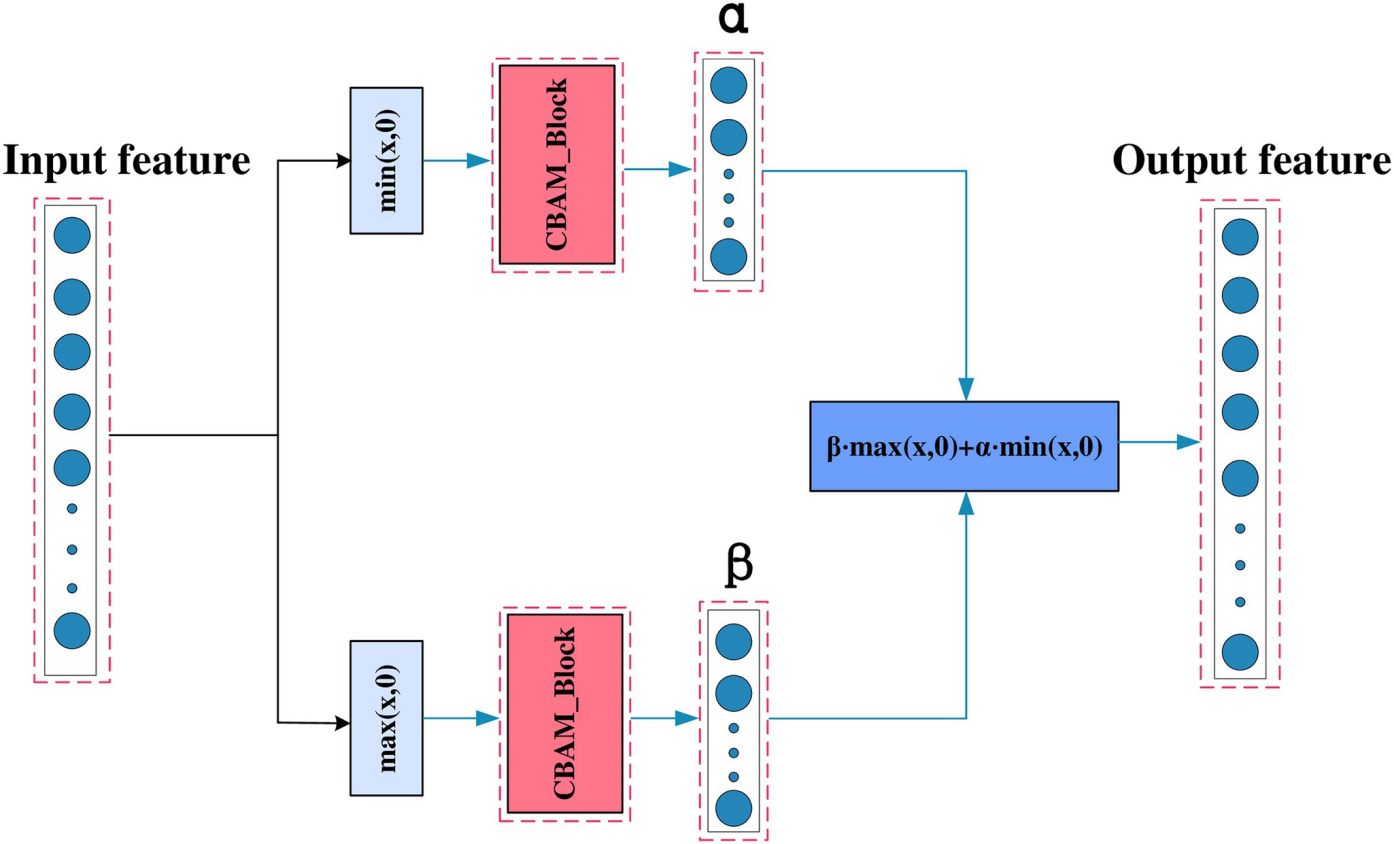
The architecture of the CGPReLU.

**Fig 10:**
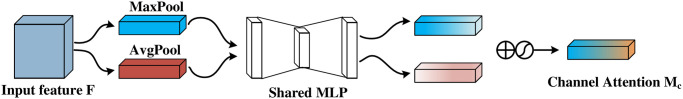
The channel attention module of CBAM.

**Fig 11:**
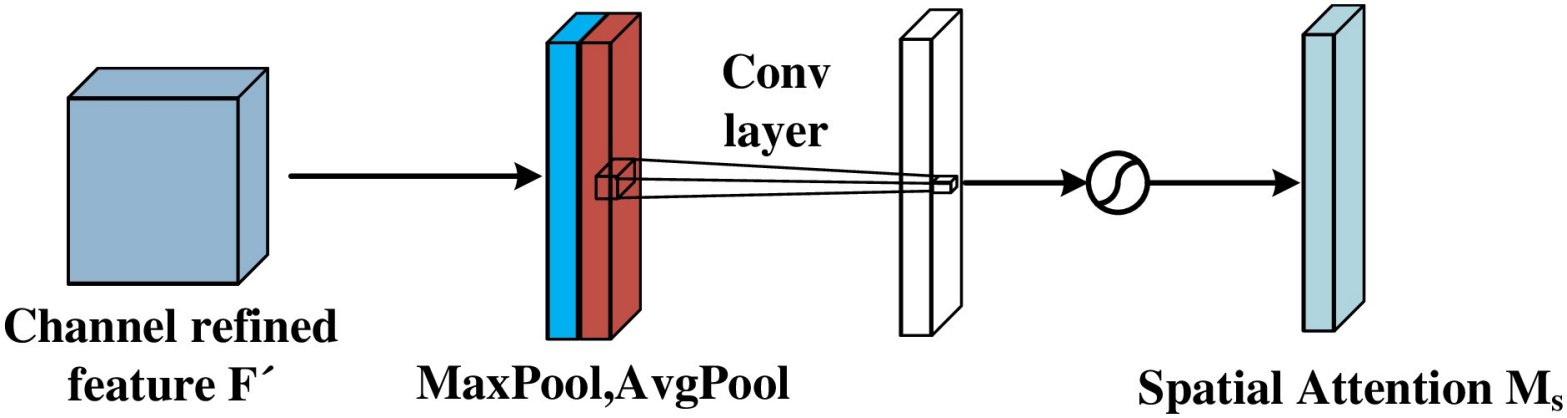
The spatial attention module of CBAM.

Where *β* is the trainable slope of the positive region. Both PReLU and GPReLU can only satisfy the nonlinear transformation of one dimensional signals. As shown in Fig 7(a) to 7(c), in order to perform multi dimensional full area adaptive nonlinear transformation on each channel, This article uses CBAM as shown in Fig 8. A new activation function, CGPReLU, is developed, which can better perform nonlinear transformation of the fault characteristics in the time frequency diagram obtain useful information to the greatest extent. As shown in Fig 7(d). CBAM is an attention module for feedforward convolutional neural networks that combines space and channels. Given an intermediate feature map, CBAM will sequentially infer the attention map from two independent dimensions, and then combine the attention map with the input feature Graph multiplication for adaptive feature optimization. Compared with the attention mechanism that only focuses on channels, it can achieve better results in feature extraction in images. The CGPReLU constructed using CBAM is shown in Fig 9. After the feature map is input into the CGPReLU module, CBAM is used to calculate the slopes of the positive and negative feature areas respectively. The channel attention module and spatial attention module in the CBAM module are shown in Figs 10 and 11 respectively.

### Depthwise separable convolutions

As the depth of the network model increases, parameters, computational requirements, and training time tend to escalate. To address this issue, this article fine tunes the residual shrinkage module and substitutes ordinary convolutions with Depthwise Separable Convolutions. Depthwise Separable Convolution comprises two steps: depthwise convolution followed by pointwise convolution. This approach helps reduce parameters, computational complexity, and training time, making the network more efficient and scalable.

**Fig 12:**
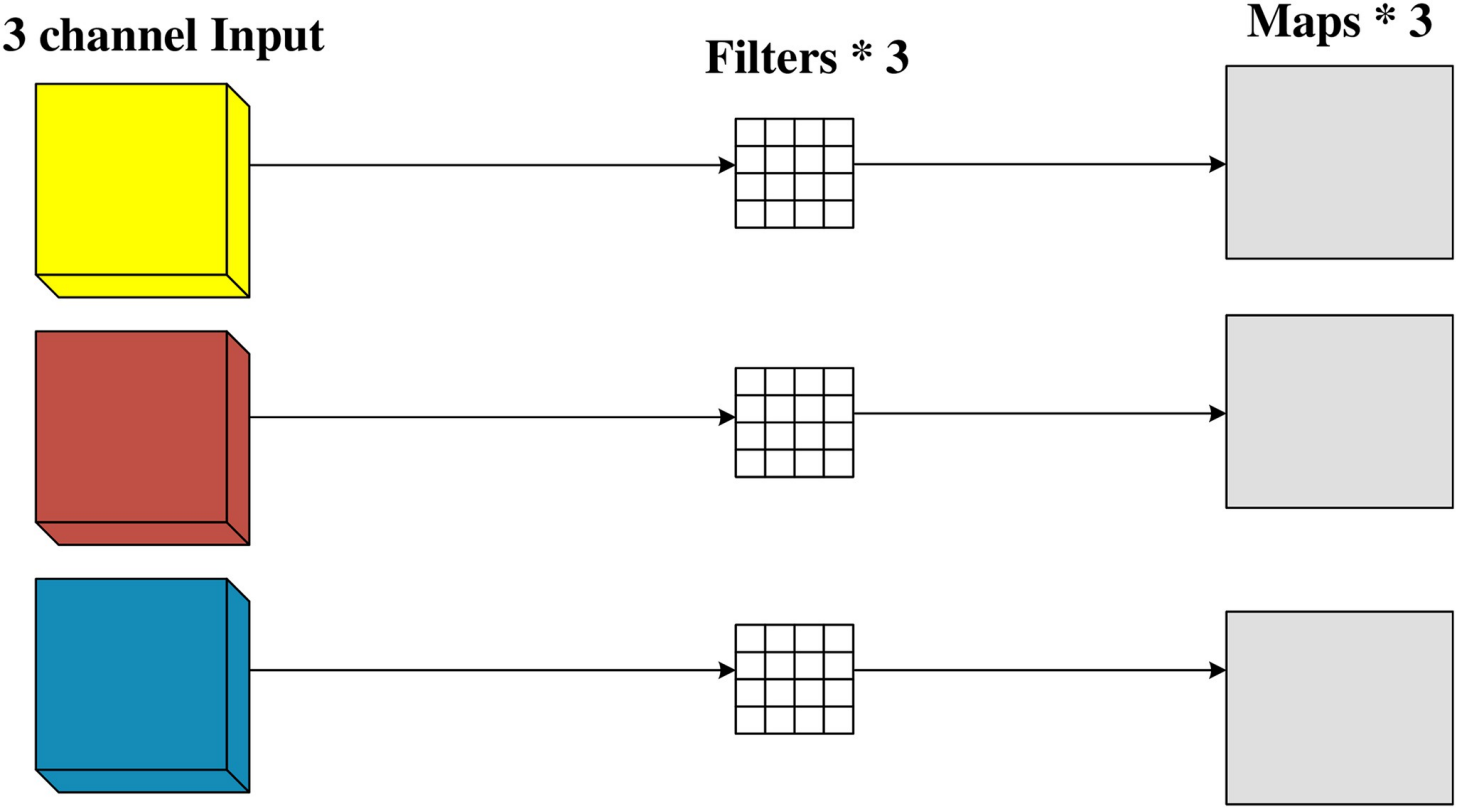
Depthwise convolution.

Depthwise convolution involves employing a convolution kernel for each channel of the input data and then concatenating the outputs of all convolution kernels to yield the final output. In contrast, depthwise separable convolution utilizes only one convolution kernel for each channel. Consequently, after the convolution operation, the output channel of a single channel remains the same as that of a normal convolution operation, which is also 1. The process of depthwise convolution is depicted in Fig 12.

**Fig 13:**
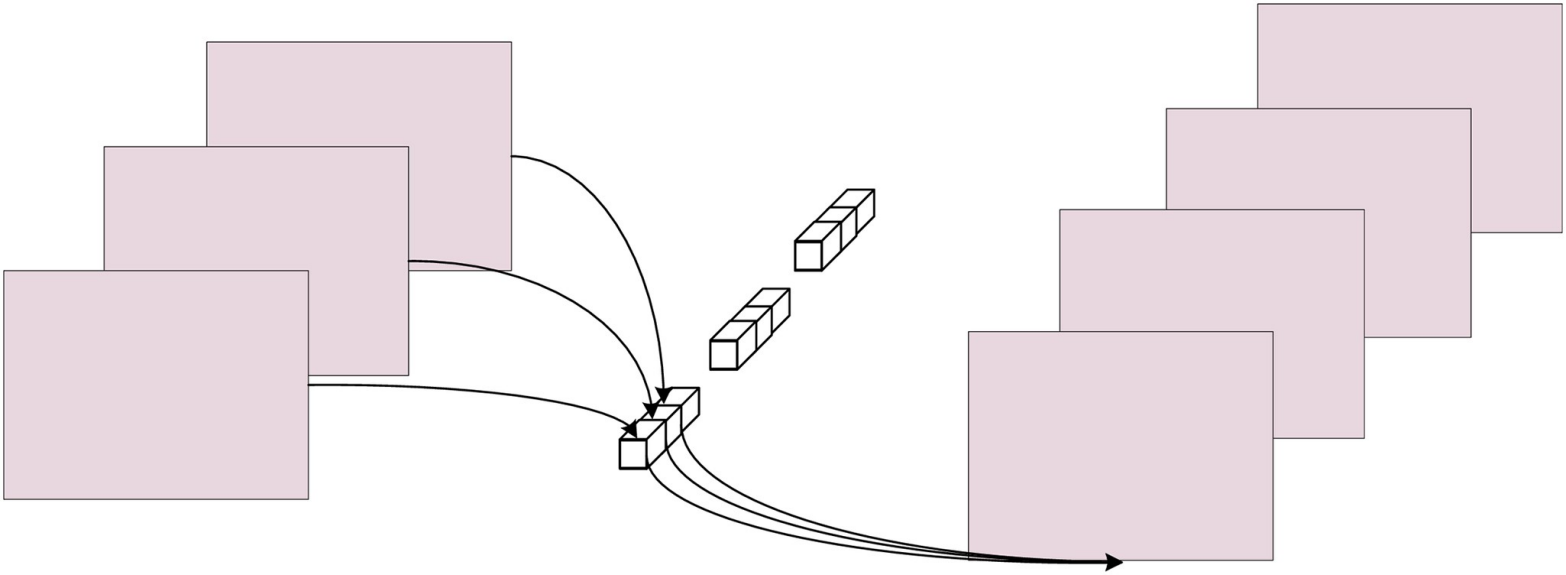
Pointwise convolution.

Pointwise convolution is actually 1 × 1 convolution. The process of pointwise convolution is shown in Fig 13. It can not only freely change the number of output channels in depthwise separable convolution, but also perform channel fusion on the output feature map.

### CCSDRSN fault diagnosis process

**Fig 14:**
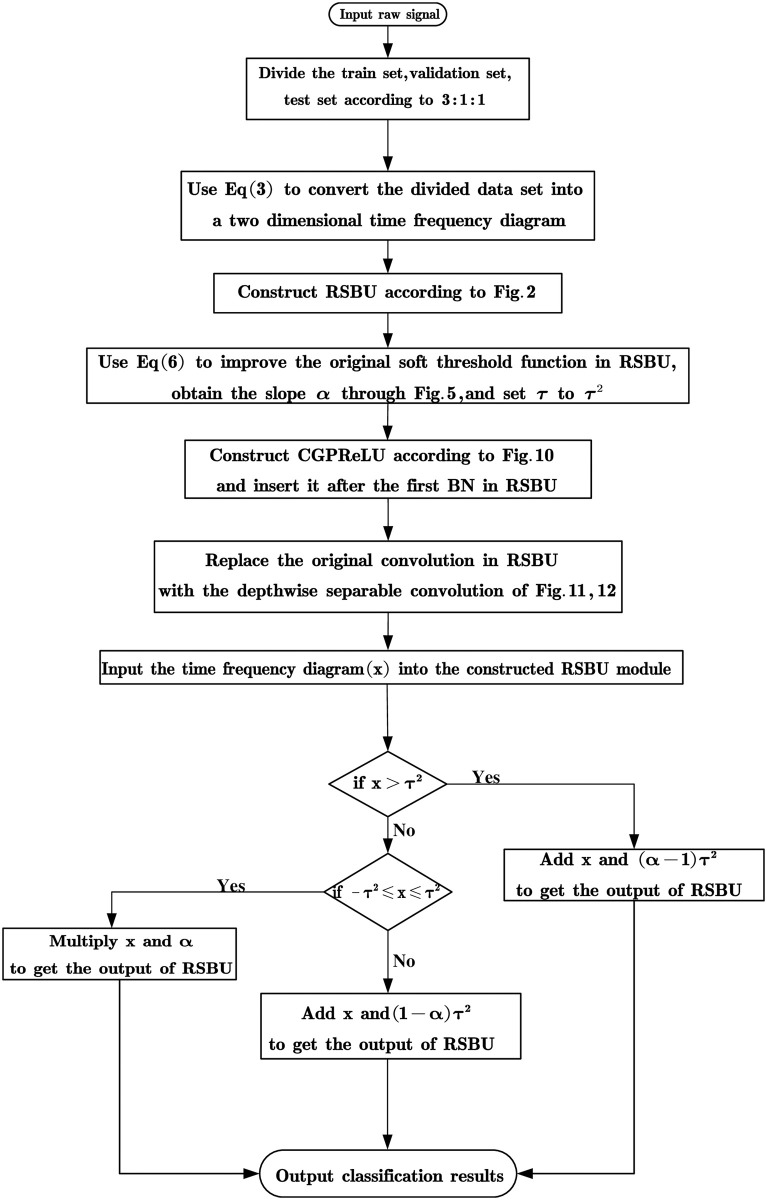
Fault diagnosis process based on CCSDRSN.

The fault diagnosis process based on CCSDRSN is as in Fig 14.

**Fig 15:**
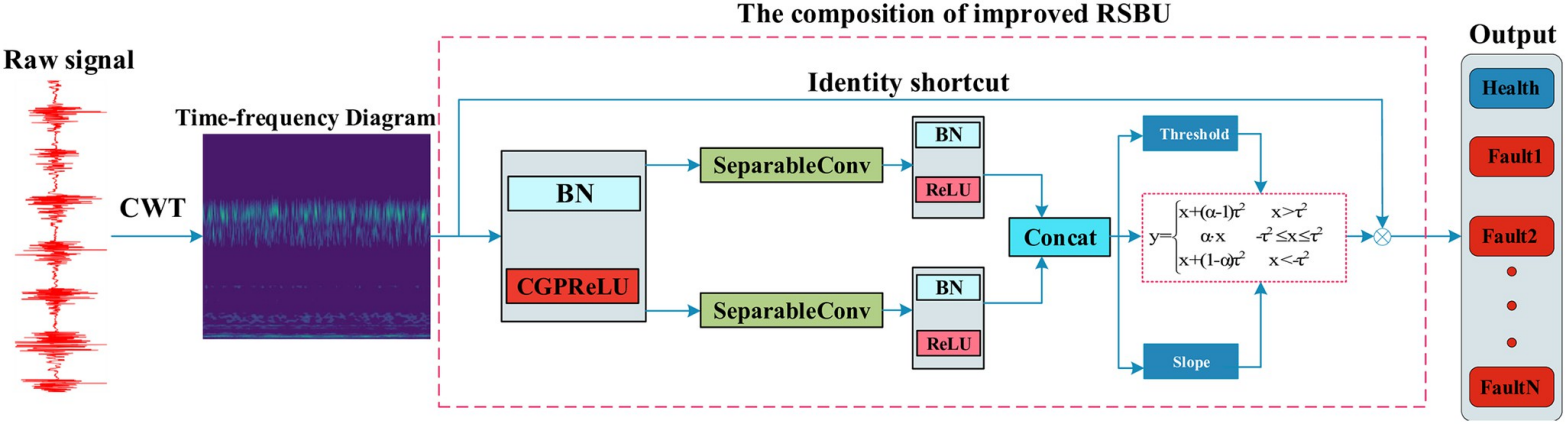
The composition of the CCSDRSN.

This article introduces a series of enhancements aimed at addressing the limitations of the DRSN fault diagnosis model, with the objective of enhancing the model’s noise resistance and refining its classification capabilities. As a result, the CCSDRSN fault diagnosis model has been developed, as depicted in Fig 15.

Initially, to address the lack of temporal information in the original one dimensional signal, the signal undergoes transformation into a time frequency diagram via continuous wavelet transform, which serves as the model’s input. Subsequently, inspired by the findings of the leaky thresholding function outlined in [29], it is noted that this function compresses the effective information of the original signal and excessively shrinks the threshold. Therefore, this study proposes an improvement to the thresholding mechanism.

Moreover, while the preprocessing of the vibration signal involves converting it into a time frequency diagram using continuous wavelet transform, the GPReLU function, as detailed in Section 2, is deemed insufficient for feature processing of the time-frequency diagram. Consequently, this article employs hybrid attention, which conducts nonlinear transformations across the entire spatial and channel domains of the input data, thereby maximizing the extraction of fault features.

Lastly, to streamline the residual shrinkage module of the model, traditional convolution operations are replaced by depthwise separable convolutions, facilitating a more efficient and lightweight model architecture.

## Experimental results

The CCSDRSN experimental environment developed in this article is Windows10 Pro (64-bit), the CPU is Intel Core i7–6700HQ, the GPU is NVIDIA GeForce GTX 960M, and the deep learning framework is TensorFlow 2.0.

### Experimental data

This study utilizes the Case Western Reserve University (CWRU) bearing dataset, specifically focusing on 0 hp, 1 hp and 2 hp cases, as a case study. Each dataset is transformed into a time frequency diagram through the application of continuous wavelet transform, and these transformed diagrams serve as the input for the enhanced DRSN model. The intent behind this approach is to validate the superior performance of the CCSDRSN network model developed in this research. Concurrently, to assess the model’s noise resistance, Gaussian white noise is introduced during data input. The detailed specifications of the bearing datasets are outlined in Table 1. The metric of Damage diameter is Miles.

**Table 1:** The description of experimental datasets.

Bearing Condition	H	OF			IF			BF		
Label	0	1	2	3	4	5	6	7	8	9
Damage diameter	0	0.007	0.014	0.021	0.007	0.014	0.021	0.007	0.014	0.021

**Fig 16:**
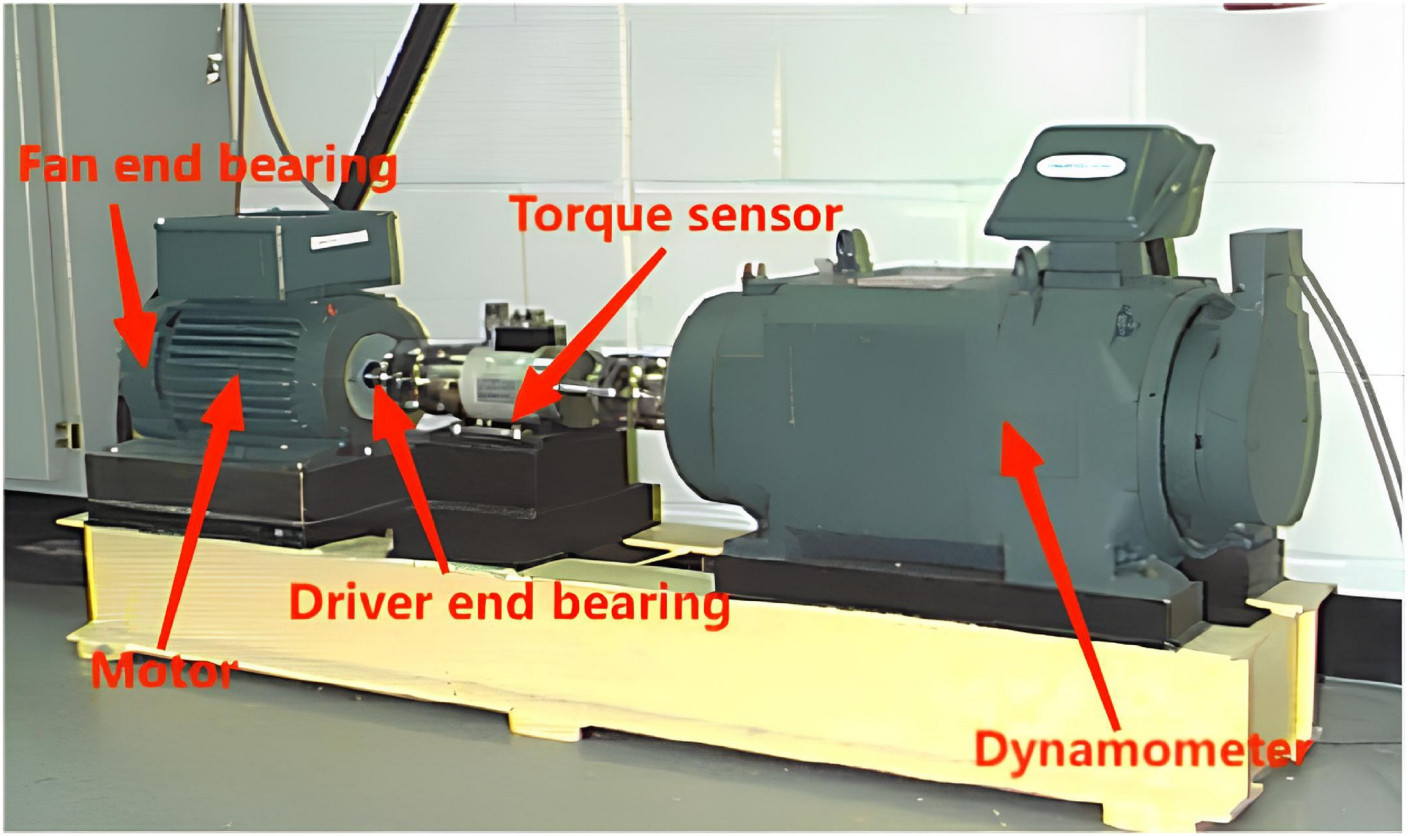
Bearing data acquisition system used by CWRU.

The acquisition device of this data set consists of a 1.5kW motor, power meter, electronic control equipment and torque sensor. The collection device diagram is shown in Fig 16. The damage locations of the rolling bearing are the rolling outer ring, inner ring, and ball. The fault conditions include outer ring failure (IF), inner ring failure (IF), and ball failure (OF). During the data collection process, electrical discharge machining (EDM) was used to cause varying degrees of damage to the inner ring, outer ring, and sphere. The fault diameters at each damage location were 0.007 inches, 0.014 inches, and 0.021 inches respectively. The fault data set contains ten bearing categories, one of which is the normal state (H). In this article, three data sets with motor loads of 0hp, 1hp and 2hp are selected to verify the classification ability of the fault diagnosis model developed in this article.

### Set noise

DRSN was proposed based on the background of strong noise. Therefore, in order to verify the performance of the model in this article, it is necessary to introduce Gaussian white noise to simulate the real operating environment of mechanical equipment. The signal-to-noise ratio (SNR) is used A common method to measure noise intensity, SNR is calculated as follows:

$$\begin{array}{c} SNR_{db}=10\;\text{log}\;(\frac{P_{signal}}{P_{noise}}) \end{array}$$ (10)

in the above formula, *P*_*signal*_ is the signal energy and *P*_*noise*_ is the noise energy. It can be seen from the signal-to-noise ratio formula that the signal-to-noise ratio is inversely proportional to the noise intensity.

In this article, Gaussian white noise processing is performed before fault data is input into the network, and verification is performed under the signal-to-noise ratio of -6db, -4db, -2db, 0db, 2db, 4db, and 6db.

### Performance comparison

- CWRU’s data set under 0hp

This article conducts a comparative analysis of the network performance of the improved Deep Residual Shrinkage Network (CCSDRSN) with traditional DRSN, CNN (Convolutional Neural Network), LSTM-CNN (Long Short Term Memory Convolutional Neural Network), and Inception network models across various noise conditions. Experimental results demonstrate that the CCSDRSN developed in this research outperforms traditional DRSN, CNN, and other network models.

**Fig 17:**
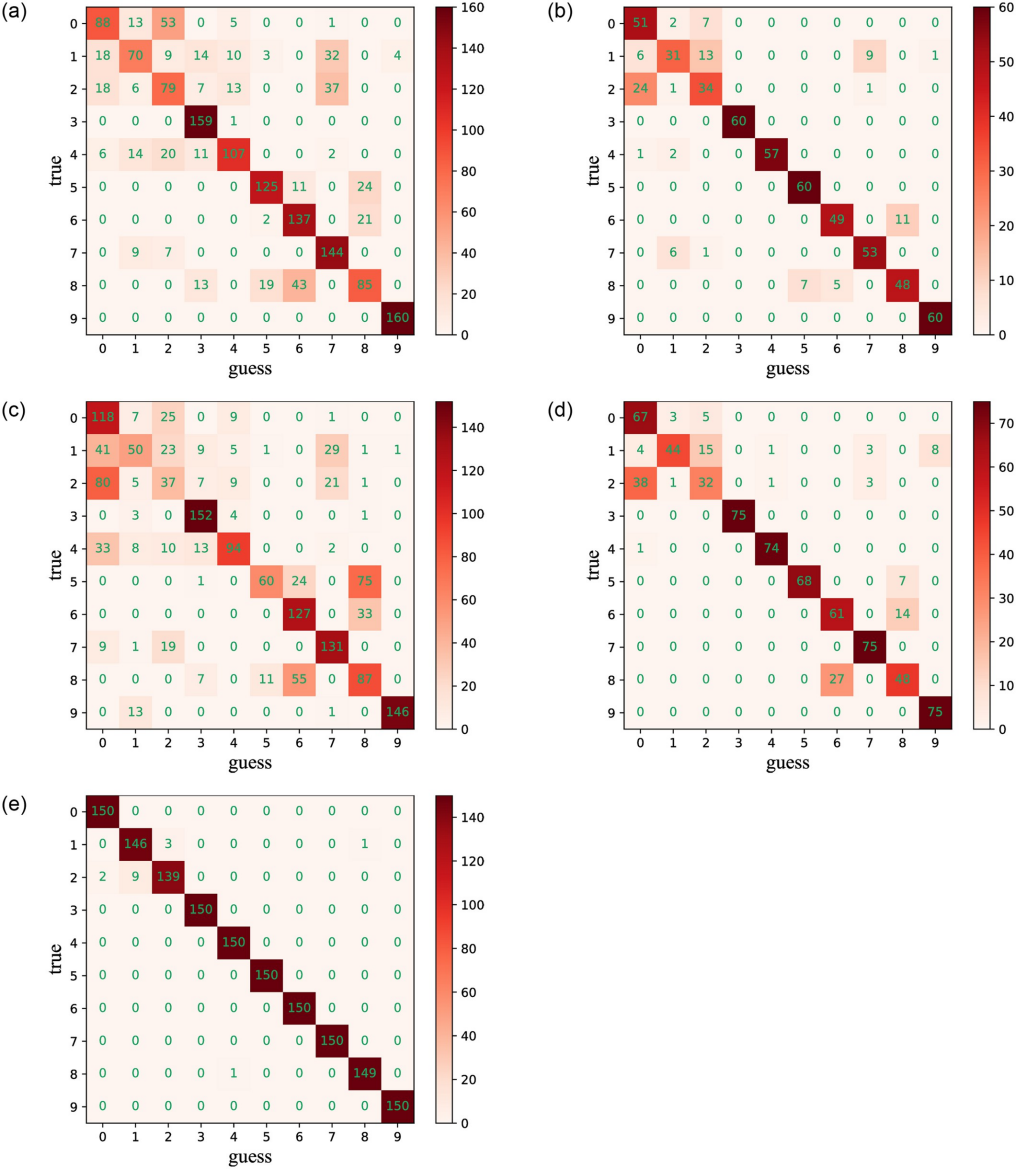
0hp confusion matrixes for different methods (SNR = -4db). (a) CNN (0hp, SNR = -4db). (b) CNN-LSTM (0hp, SNR = -4db). (c) DRSN (0hp, SNR = -4db). (d) Inception (0hp, SNR = -4db). (e) CCSDRSN (0hp, SNR = -4db).

Regarding fault diagnosis accuracy, it is confirmed that the CCSDRSN model structure enhances diagnosis accuracy and reduces loss rates. As depicted in Fig 17, the confusion matrix of different network models under an SNR of -4dB is presented. The abscissa represents the Guess Label, corresponding to the predicted category, while the ordinate represents the True Label, corresponding to the real category. The values on the diagonal of the matrix indicate the number of correctly predicted samples. The darker the color in the diagonal, the higher the number of predicted samples.

To provide a visually compelling representation of the classification performance of CCSDRSN, this article utilizes the t-SNE (t-Distributed Stochastic Neighbor Embedding) technique. Originally introduced in 2008 by Laurens van der Maaten and Geoffrey Hinton, t-SNE is a nonlinear dimensionality reduction algorithm specifically designed for machine learning. It excels at condensing high dimensional data into two or three dimensions, facilitating more accessible visualization.

**Fig 18:**
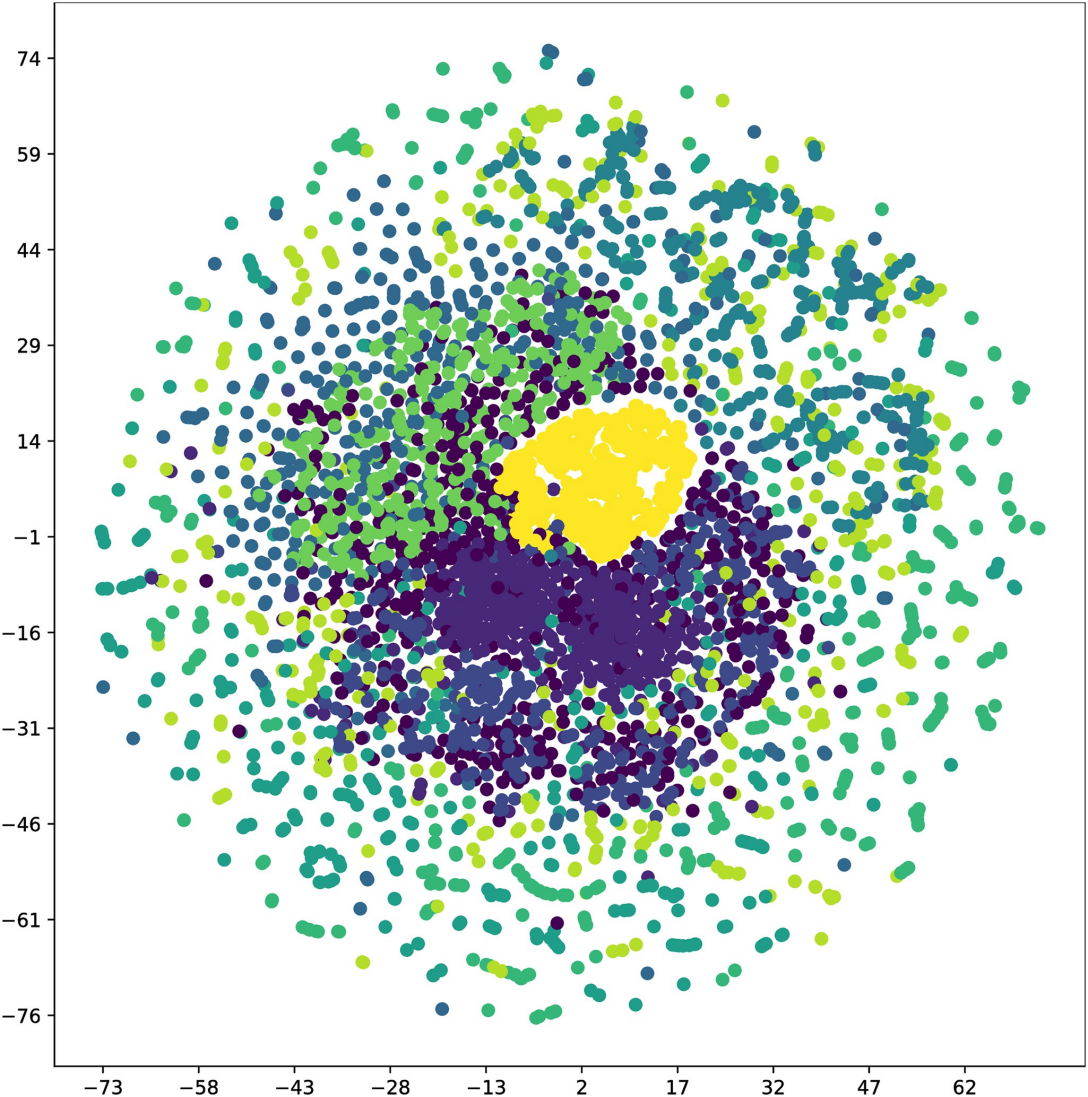
t-SNE visualization of original data.

Following the collection of the original fault vibration signals, t-SNE is initially applied for dimensionality reduction and visualization, as depicted in Fig 18. The determination of the horizontal and vertical coordinates is rooted in the similarity of sample points within the high dimensional space and the corresponding distances in the low dimensional space. Consequently, samples with the same labels exhibit minimal intra class separation, while samples with different labels are distinctly separated. Prior to classification, most raw data samples projected onto a two dimensional plane appear unordered, making it challenging to discern distinct data types.

**Fig 19:**
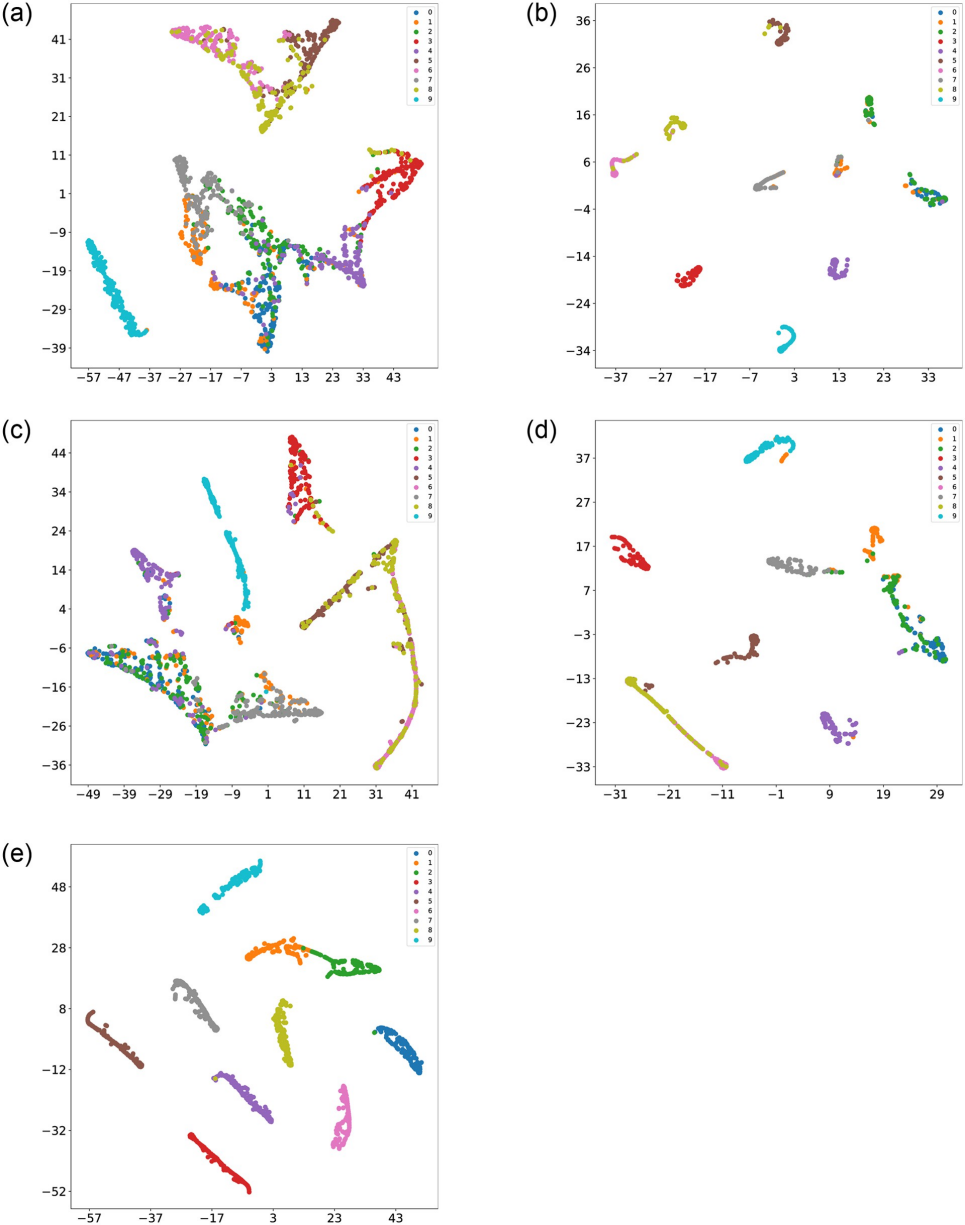
Visualization of classification results for different methods. (a) CNN (0hp, SNR = -4db). (b) CNN-LSTM (0hp, SNR = -4db). (c) DRSN (0hp, SNR = -4db). (d) Inception (0hp, SNR = -4db). (e) CCSDRSN (0hp, SNR = -4db).

The results after classification are illustrated in Fig 19. Apart from CCSDRSN developed in this article, there are varying degrees of mixing, overlap, and other issues in the data classification of other network models. It can be concluded that CCSDRSN demonstrates robust classification accuracy even in high intensity noise conditions.

**Fig 20:**
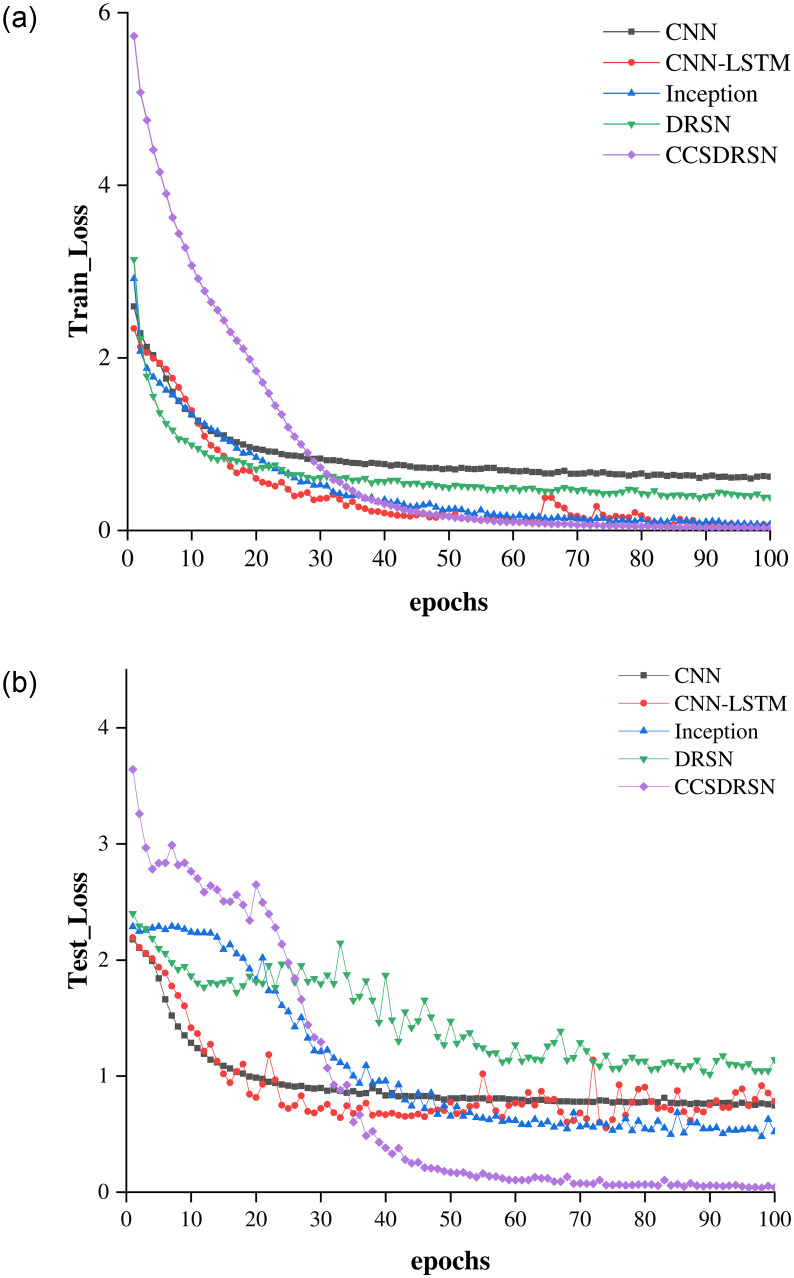
Training and test loss for different methods. (a) Training loss (SNR = -4 db,0hp). (b) Test loss (SNR = -4 db, 0hp).

The loss rates for the training and test sets of DRSN, CNN, CNN-LSTM, Inception, and the CCSDRSN developed in this article are presented in Fig 20, where the x axis denotes the number of model training iterations, and the y axis indicates the loss value. Under identical conditions, the CCSDRSN exhibits significantly lower losses for both the training and test sets compared to other network models. Particularly noteworthy is the CCSDRSN’s ability to minimize loss rapidly on the test set. The final losses of different methods on the training set and test set are shown in Table 2, This enhanced performance can be attributed to the improved threshold function detailed in this study, which addresses the issue of insufficient effective information extraction due to excessive threshold shrinkage. The adaptive threshold slope is designed to retain as much useful information as possible. Additionally, the novel CGPReLU activation function enables multidimensional nonlinear transformations of the global fault characteristic regions on the time frequency diagram, facilitating the differentiation of fault signal characteristics from normal signals and bolstering the model’s classification capabilities. Consequently, the CCSDRSN demonstrates a swifter convergence rate and superior classification performance under -4dB conditions for 0HP in the CWRU dataset.

**Table 2:** Final loss of training set and test set under different methods(0hp -4db).

different dataset	CNN	CNN-LSTM	Inception	DRSN	CCSDRSN
Training Loss	0.621	0.061	0.067	0.385	0.025
Test Loss	0.745	0.783	0.518	1.143	0.040

**Fig 21:**
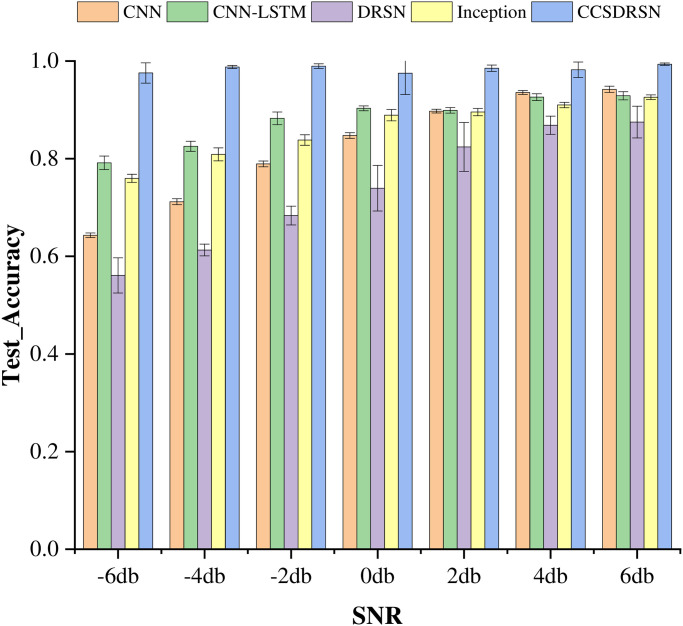
Test accuracy of different methods under different noise intensities(0hp).

The average test accuracy serves as a critical metric for evaluating the performance of the models, Standard deviation (standard deviation for short) is the square root of the variance of the sample mean. It reflects the degree of dispersion among individuals within a group. In this study, the mean and standard deviation were adopted as methods to evaluate the diagnostic accuracy of various models. as depicted in Fig 21. Notably, among the five models, the CCSDRSN developed in this study stands out as the optimal performer across a range of signal-to-noise ratios from -6dB to 6dB. The traditional DRSN model, in comparison to other network models, exhibits inferior performance. This is attributed to the fact that the current study only incorporates a single layer of RSBU for noise reduction, without stacking multiple layers. It is noteworthy that both the CNN-LSTM and Inception network models demonstrate commendable performance. However, the CCSDRSN network structure remains robust in maintaining high classification capabilities even under a high noise environment, highlighting its capacity to effectively suppress noise. Thus, the network model presented in this article has the potential to significantly enhance the accuracy of fault diagnosis.

### Performance comparison

- CWRU’s data set under 1hp

**Fig 22:**
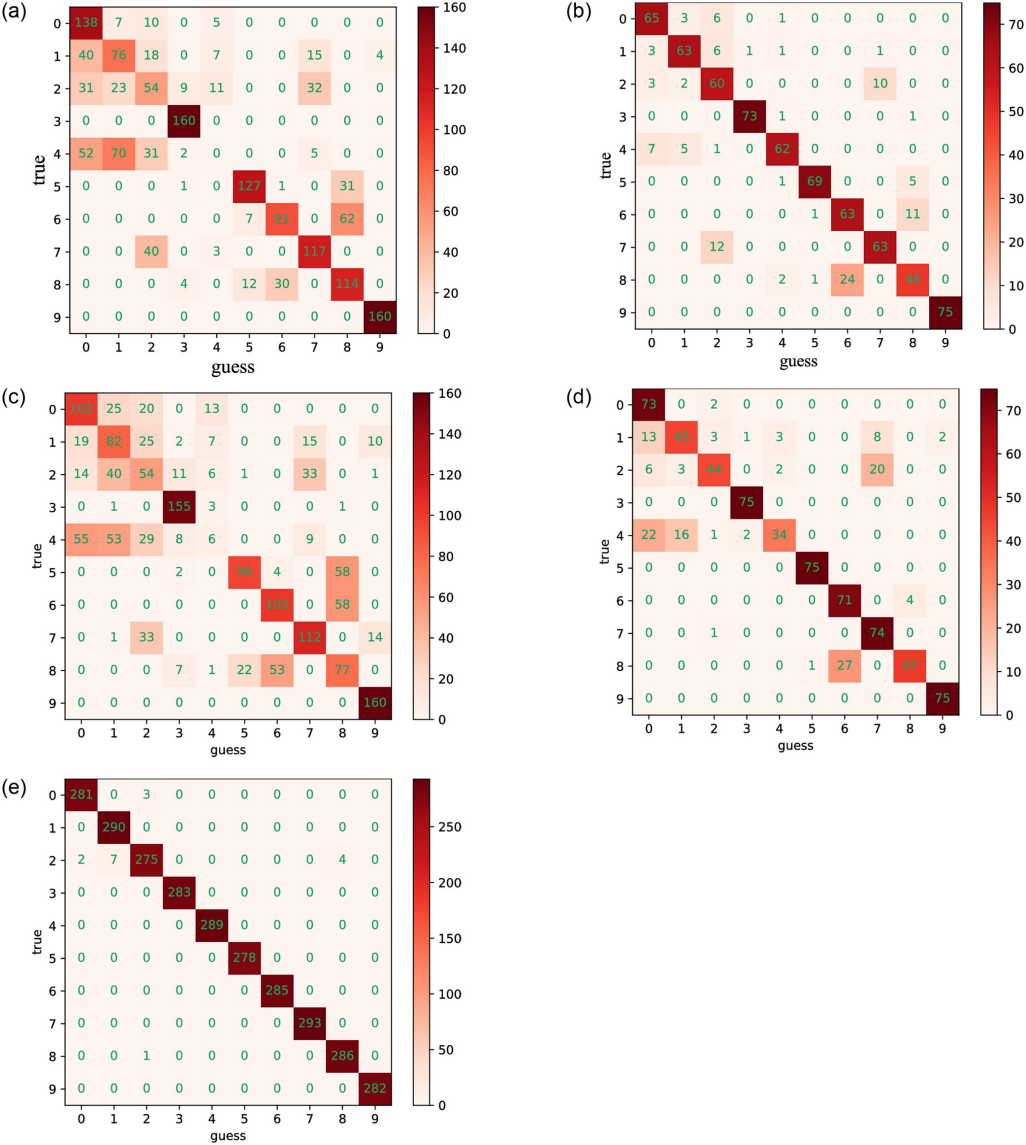
1hp Confusion matrixes for different methods (SNR = -4db). (a) CNN (1hp, SNR = -4db). (b) CNN-LSTM (1hp, SNR = -4db). (c) DRSN (1hp, SNR = -4db). (d) Inception (1hp, SNR = -4db). (e) CCSDRSN (1hp, SNR = -4db).

**Fig 23:**
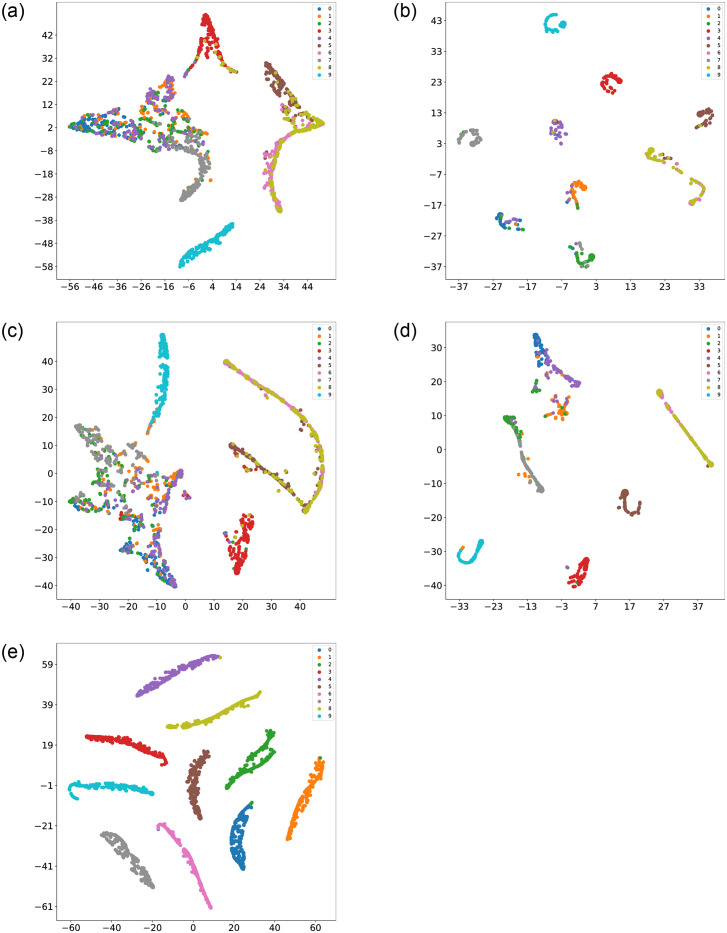
Visualization of classification results for different methods. (a) CNN (1hp, SNR = -4db). (b) CNN-LSTM (1hp, SNR = -4db). (c) DRSN (1hp, SNR = -4db). (d) Inception (1hp, SNR = -4db). (e) CCSDRSN (1hp, SNR = -4db).

In this section, the 1hp dataset of CWRU is used to further verify the effectiveness of CCSDRSN. Taking the generalization ability of the model under a signal-to-noise ratio of -4dB as an example, it can be seen from the confusion matrix plotting results of Fig 22(e) and the classification result visualization of Fig 23(e) that, except for a very small number of samples, the vast majority of samples are accurately classified, with a diagnostic accuracy of more than 98%.

**Fig 24:**
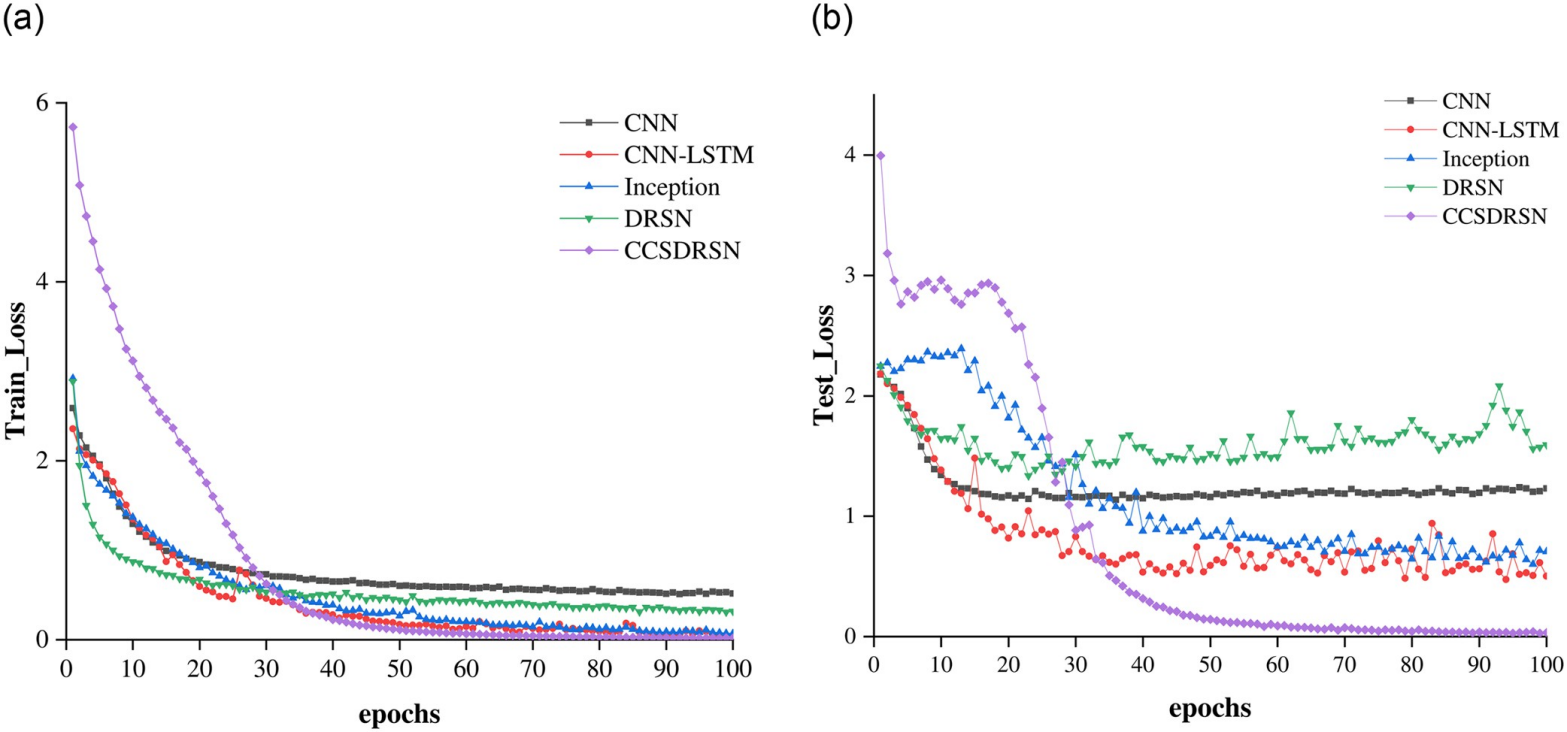
Training and test loss for different methods. (a) Training loss (SNR = -4 db, 1hp). (b) Test loss (SNR = -4 db, 1hp).

The loss rates for the training and test sets of DRSN, CNN, CNN-LSTM, Inception, and the CCSDRSN developed in this article are presented in Fig 24, Under identical conditions, The loss of CCSDRSN on both training and test sets is still lower than that of other network models. The final losses of different methods on the training set and test set are shown in Table 3.

**Table 3:** Final loss of training set and test set under different methods(1hp -4db).

different dataset	CNN	CNN-LSTM	Inception	DRSN	CCSDRSN
Training Loss	0.517	0.027	0.066	0.318	0.017
Test Loss	1.230	0.501	0.704	1.593	0.031

**Fig 25:**
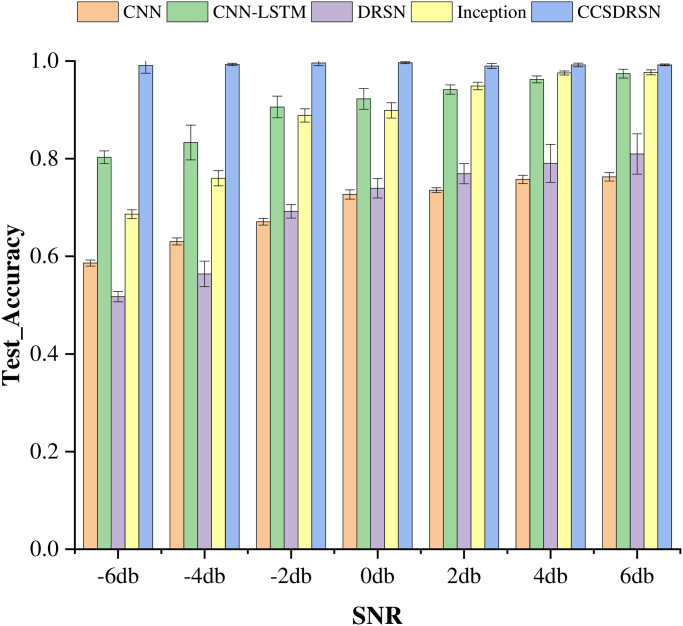
Test accuracy of different methods under different noise intensities(1hp).

This section still uses the average test accuracy method to evaluate model performance. as depicted in Fig 25.among the five models, The CCSDRSN proposed in this paper still performs best in the -6dB 6dB signal-to-noise ratio range. From the results of the low diagnostic accuracy of the CNN model, it can be seen that because the one dimensional signal contains time series information, the CNN-LSTM model combined with LSTM still has strong generalization ability.

- CWRU’s data set under 2hp

**Fig 26:**
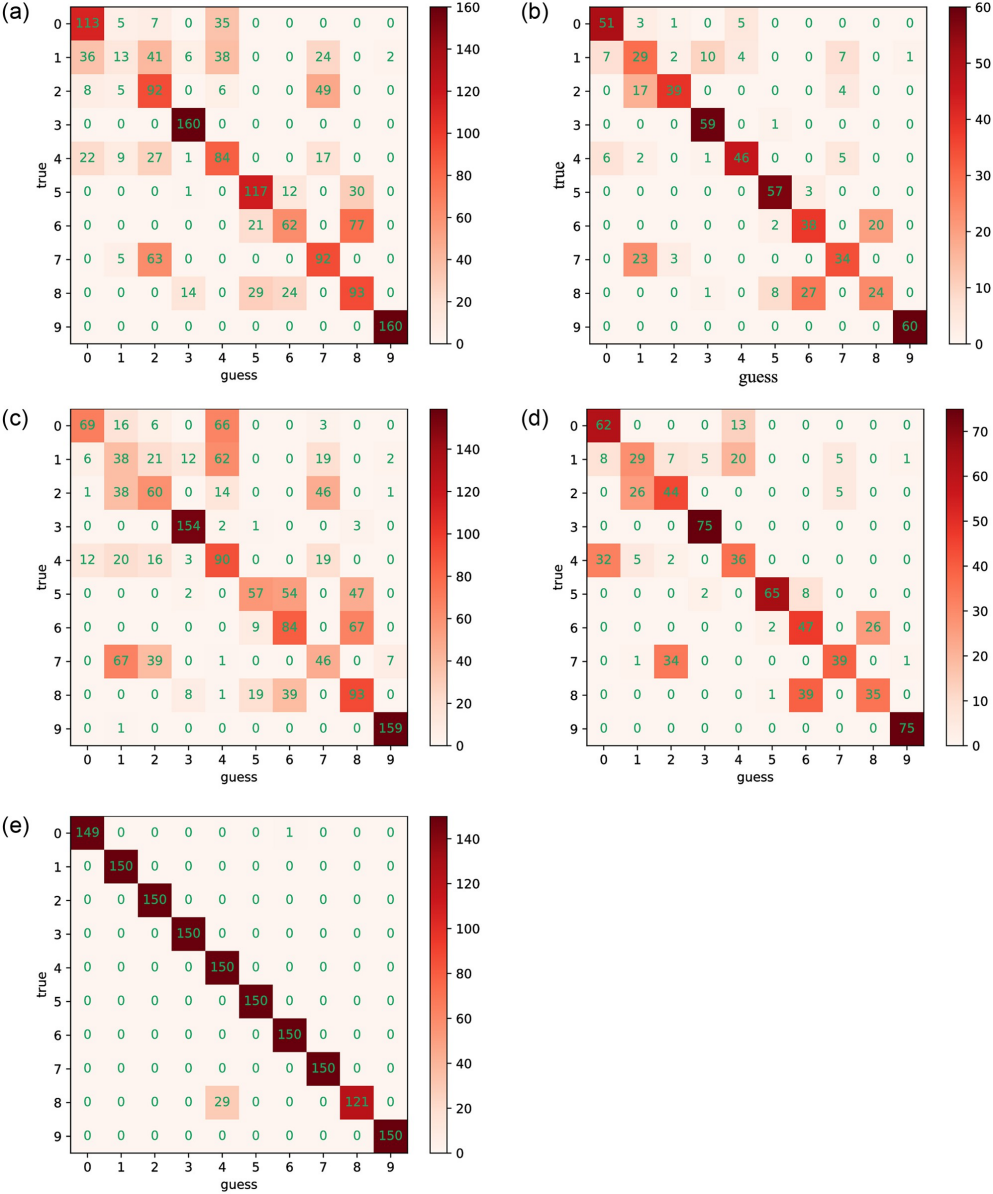
2hp confusion matrixes for different methods (SNR = -6db). (a) CNN (2hp, SNR = -6db). (b) CNN-LSTM (2hp, SNR = -6db). (c) DRSN (2hp, SNR = -6db). (d) Inception (2hp, SNR = -6db). (e) CCSDRSN (2hp, SNR = -6db).

**Fig 27:**
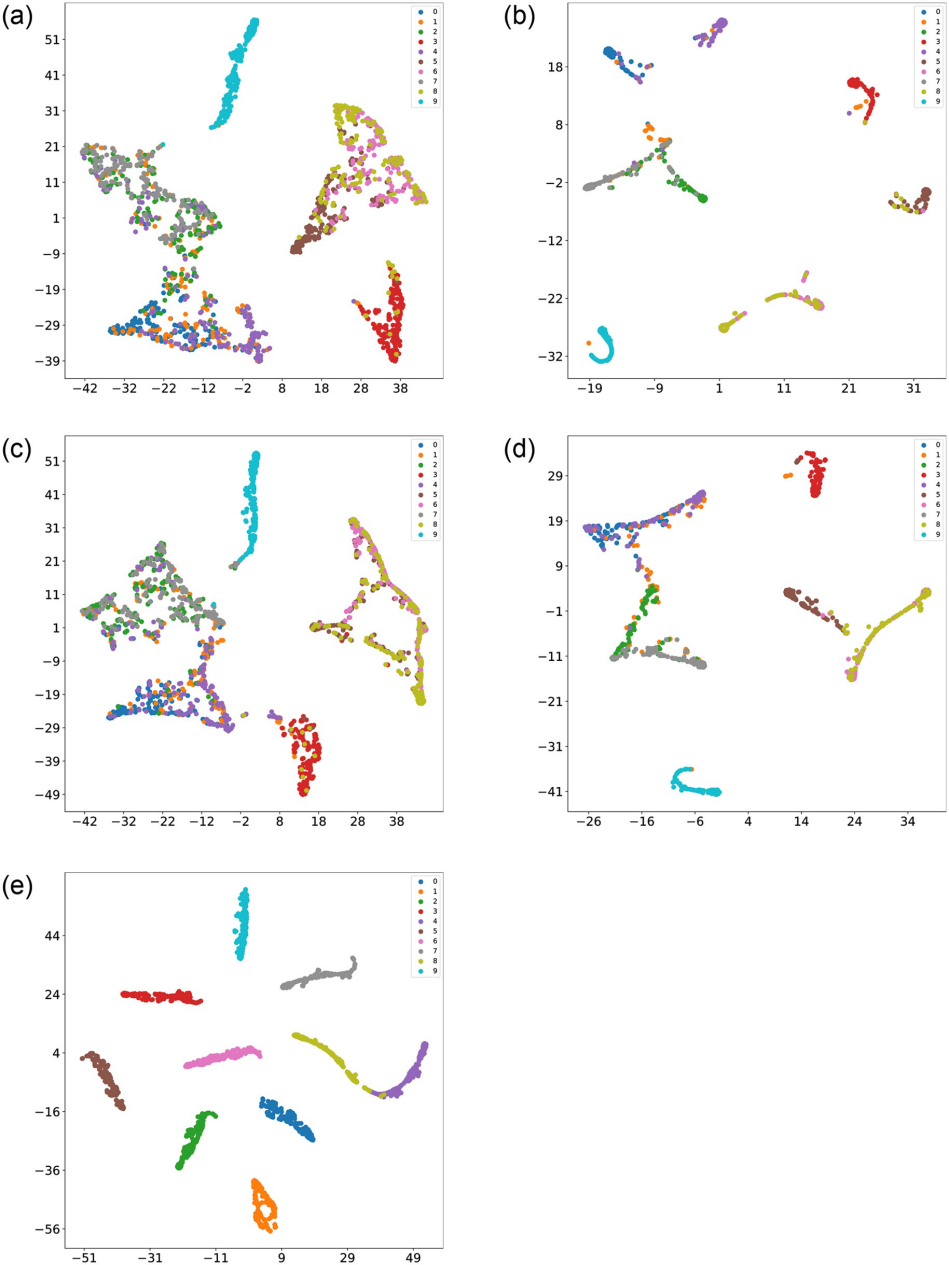
Visualization of classification results for different methods. (a) CNN (2hp, SNR = -6db). (b) CNN-LSTM (2hp, SNR = -6db). (c) DRSN (2hp, SNR = -6db) (d) Inception (2hp, SNR = -6db). (e) CCSDRSN (2hp, SNR = -6db).

In this section, the robustness of CCSDRSN is further substantiated using the 2hp dataset from CWRU under a signal-to-noise ratio of -6dB. The network model’s classification performance under these conditions is illustrated in Figs 26 and 27. From the confusion matrix plotting results in Fig 26(e) and the Visualization of classification results in Fig 27(e), we can see that, it is evident that the vast majority of samples were accurately classified, with only a few exceptions, resulting in a diagnostic accuracy exceeding 99%.

**Fig 28:**
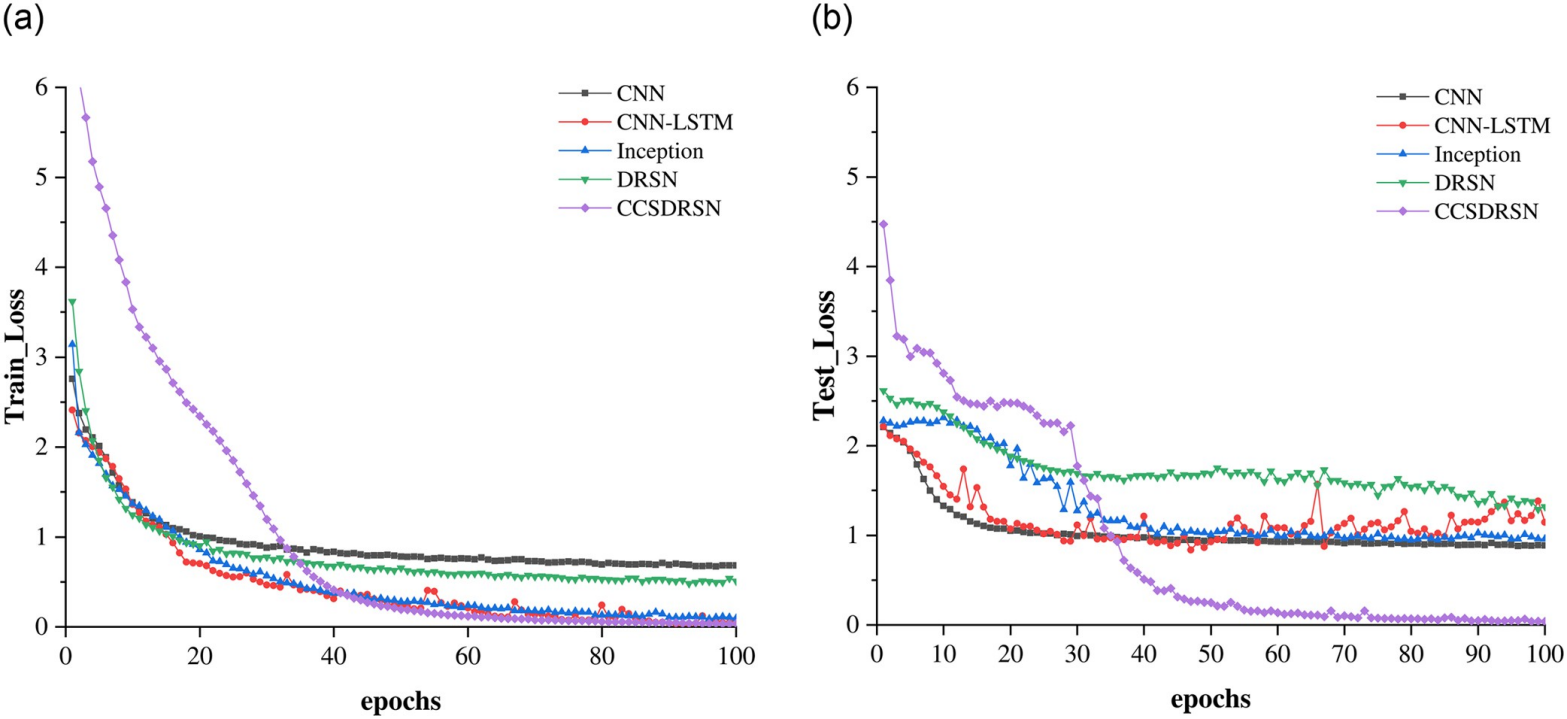
Training loss and test loss for different methods (SNR = -6 dB, 2hp). (a) Training loss. (b) Test loss.

It can be seen from the loss rate of the training set in Fig 28(a) that although the network model has the largest loss at the beginning, as the number of iterations increases, its convergence speed gradually accelerates, and it reaches the lowest loss rate after 40 iterations. The same performance is more obvious in the loss rate of the test set. As shown in Fig 28(b), the loss rate of the test set has reached the lowest after 35 iterations, and finally approaches zero. As can be seen from Table 4, CCSDRSN is the lowest in terms of final losses on both the test set and the training set.

**Table 4:** Final loss of training set and test set under different methods (2hp -6db).

different dataset	CNN	CNN-LSTM	Inception	DRSN	CCSDRSN
Training Loss	0.683	0.077	0.095	0.508	0.036
Test Loss	0.887	1.145	0.956	1.321	0.037

**Fig 29:**
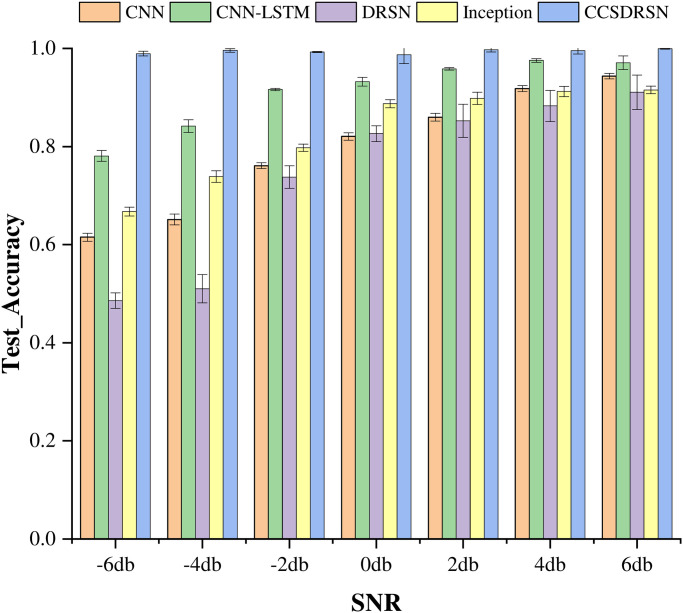
Test accuracy of different methods under different noise intensities(2hp).

In order to underscore the high diagnostic accuracy of CCSDRSN at 2hp, this section employs the method of average test accuracy and standard deviation to generate a histogram, as depicted in Fig 29. Across various signal-to-noise ratios, the model developed in this article consistently achieves accuracy levels above 99%. Particularly noteworthy is the performance advantage of CCSDRSN under a signal-to-noise ratio of -6dB. When noise intensity is low, the CNN network model under 2hp appears to learn more fault features compared to the network under 1hp. Consequently, under the condition of 6dB, its classification accuracy can reach 94%. Additionally, the combination of CNN and LSTM shows potential in enhancing fault diagnosis accuracy under weak noise conditions.

## Conclusions

This article proposes a fault diagnosis model that integrates continuous wavelet transform and an enhanced deep residual shrinkage network. Continuous wavelet transform is utilized to extract valuable time frequency information from the signal. To address the issues of insufficient effective information extraction and excessive threshold shrinkage in the original soft threshold of DRSN, an improved soft threshold function is introduced. Additionally, a new activation function, CGPReLU, is developed for the data preprocessing operation, converting the original one dimensional vibration signal into a time frequency diagram. This activation function leverages CBAM to nonlinearly transform fault information contained in the time-frequency diagram from both spatial and channel perspectives. Furthermore, the depth residual shrinkage module is improved by replacing ordinary convolution with a depthwise separable convolution module.

Empirical results demonstrate a 5.17% reduction in model training time and approximately a 0.39% reduction in the number of parameters, thereby enhancing the computational efficiency of the model.

To comprehensively assess the performance of this model, comparisons are made with DRSN, CNN, CNN-LSTM, and Inception network models using the CWRU dataset. Evaluation methods include data dimensionality reduction visualization, confusion matrix analysis, test set loss rate, and average test accuracy. The results indicate that the model developed in this article exhibits strong noise resistance and high classification accuracy, as evidenced by various evaluation metrics.

While this article surpasses most models in diagnostic accuracy, it lags behind many in terms of time consumption. However, this serves as an area for potential improvement in future research endeavors. It’s essential to address this issue to enhance the model’s overall efficiency and applicability in real world scenarios. Further optimization strategies could focus on streamlining computational processes, reducing unnecessary complexity, and exploring more efficient algorithms or architectures. By striking a balance between accuracy and computational efficiency, future iterations of the model can better meet the demands of practical implementation in fault diagnosis systems.

## Supporting information

S1 Data (ZIP)

## References

[1] Chen X, Zhang B, Gao D. Bearing fault diagnosis base on multi-scale CNN and LSTM model. Journal of Intelligent Manufacturing. 2021;32:971–987. doi: 10.1007/s10845-020-01600-2

[2] Chang C, Wang Q, Jiang J, Jiang Y, Wu T. Voltage fault diagnosis of a power battery based on wavelet time-frequency diagram. Energy. 2023;278:127920. doi: 10.1016/j.energy.2023.127920

[3] Zhang Y, Zhou T, Huang X, Cao L, Zhou Q. Fault diagnosis of rotating machinery based on recurrent neural networks. Measurement. 2021;171:108774. doi: 10.1016/j.measurement.2020.108774

[4] Zhao M, Fu X, Zhang Y, Meng L, Tang B. Highly imbalanced fault diagnosis of mechanical systems based on wavelet packet distortion and convolutional neural networks. Advanced Engineering Informatics. 2022;51:101535. doi: 10.1016/j.aei.2022.101535

[5] Cheng Y, Lin M, Wu J, Zhu H, Shao X. Intelligent fault diagnosis of rotating machinery based on continuous wavelet transform-local binary convolutional neural network. Knowledge-Based Systems. 2021;216:106796. doi: 10.1016/j.knosys.2021.106796

[6] Zhao H, Liu J, Chen H, Chen J, Li Y, Xu J, et al. Intelligent diagnosis using continuous wavelet transform and gauss convolutional deep belief network. IEEE Transactions on Reliability. 2022;.

[7] Chen R, Huang X, Yang L, Xu X, Zhang X, Zhang Y. Intelligent fault diagnosis method of planetary gearboxes based on convolution neural network and discrete wavelet transform. Computers in industry. 2019;106:48–59. doi: 10.1016/j.compind.2018.11.003

[8] Ni Q, Ji J, Feng K. Data-driven prognostic scheme for bearings based on a novel health indicator and gated recurrent unit network. IEEE Transactions on Industrial Informatics. 2022;19(2):1301–1311. doi: 10.1109/TII.2022.3169465

[9] Feng K, Ji J, Zhang Y, Ni Q, Liu Z, Beer M. Digital twin-driven intelligent assessment of gear surface degradation. Mechanical Systems and Signal Processing. 2023;186:109896. doi: 10.1016/j.ymssp.2022.109896

[10] Wu J, He D, Li J, Miao J, Li X, Li H, et al. Temporal multi-resolution hypergraph attention network for remaining useful life prediction of rolling bearings. Reliability Engineering & System Safety. 2024;247:110143. doi: 10.1016/j.ress.2024.110143

[11] Zhao Z, Zhang Q, Yu X, Sun C, Wang S, Yan R, et al. Applications of unsupervised deep transfer learning to intelligent fault diagnosis: A survey and comparative study. IEEE Transactions on Instrumentation and Measurement. 2021;70:1–28. doi: 10.1109/TIM.2021.312601933776080

[12] Chen X, Yang R, Xue Y, Huang M, Ferrero R, Wang Z. Deep transfer learning for bearing fault diagnosis: A systematic review since 2016. IEEE Transactions on Instrumentation and Measurement. 2023;.

[13] Ding Y, Jia M, Zhuang J, Cao Y, Zhao X, Lee CG. Deep imbalanced domain adaptation for transfer learning fault diagnosis of bearings under multiple working conditions. Reliability Engineering & System Safety. 2023;230:108890. doi: 10.1016/j.ress.2022.108890

[14] Li C, Li S, Wang H, Gu F, Ball AD. Attention-based deep meta-transfer learning for few-shot fine-grained fault diagnosis. Knowledge-Based Systems. 2023;264:110345. doi: 10.1016/j.knosys.2023.110345

[15] He K, Zhang X, Ren S, Sun J. Deep residual learning for image recognition. In: Proceedings of the IEEE conference on computer vision and pattern recognition; 2016. p. 770–778.

[16] Liang P, Wang W, Yuan X, Liu S, Zhang L, Cheng Y. Intelligent fault diagnosis of rolling bearing based on wavelet transform and improved ResNet under noisy labels and environment. Engineering Applications of Artificial Intelligence. 2022;115:105269. doi: 10.1016/j.engappai.2022.105269

[17] Ma S, Chu F, Han Q. Deep residual learning with demodulated time-frequency features for fault diagnosis of planetary gearbox under nonstationary running conditions. Mechanical Systems and Signal Processing. 2019;127:190–201. doi: 10.1016/j.ymssp.2019.02.055

[18] Ni Q, Ji J, Halkon B, Feng K, Nandi AK. Physics-Informed Residual Network (PIResNet) for rolling element bearing fault diagnostics. Mechanical Systems and Signal Processing. 2023;200:110544. doi: 10.1016/j.ymssp.2023.110544

[19] Zhao M, Zhong S, Fu X, Tang B, Dong S, Pecht M. Deep residual networks with adaptively parametric rectifier linear units for fault diagnosis. IEEE Transactions on Industrial Electronics. 2020;68(3):2587–2597. doi: 10.1109/TIE.2020.2972458

[20] Zhao M, Zhong S, Fu X, Tang B, Pecht M. Deep residual shrinkage networks for fault diagnosis. IEEE Transactions on Industrial Informatics. 2019;16(7):4681–4690. doi: 10.1109/TII.2019.2943898

[21] Yang J, Gao T, Jiang S, Li S, Tang Q. Fault diagnosis of rotating machinery based on one-dimensional deep residual shrinkage network with a wide convolution layer. Shock and Vibration. 2020;2020:1–12. doi: 10.1155/2020/3769206

[22] Hu H, Ma X, Shang Y. A novel method for transformer fault diagnosis based on refined deep residual shrinkage network. IET Electric Power Applications. 2022;16(2):206–223. doi: 10.1049/elp2.12147

[23] Salimy A, Mitiche I, Boreham P, Nesbitt A, Morison G. Dynamic noise reduction with deep residual shrinkage networks for online fault classification. Sensors. 2022;22(2):515. doi: 10.3390/s22020515 35062476 PMC8781998

[24] Pei X, Dong S, Tang B, Pan X. Bearing running state recognition method based on feature-to-noise energy ratio and improved deep residual shrinkage network. IEEE/ASME Transactions on Mechatronics. 2021;27(5):3660–3671. doi: 10.1109/TMECH.2021.3120755

[25] Tong J, Tang S, Wu Y, Pan H, Zheng J. A fault diagnosis method of rolling bearing based on improved deep residual shrinkage networks. Measurement. 2023;206:112282. doi: 10.1016/j.measurement.2022.112282

[26] Zhang Z, Li H, Chen L, Han P. Rolling bearing fault diagnosis using improved deep residual shrinkage networks. Shock and Vibration. 2021;2021:1–11.

[27] Zhang Z, Li H, Chen L. Deep residual shrinkage networks with self-adaptive slope thresholding for fault diagnosis. In: 2021 7th International Conference on Condition Monitoring of Machinery in Non-Stationary Operations (CMMNO). IEEE; 2021. p. 236–239.

[28] Zhang Z, Zhang C, Zhang X, Chen L, Shi H, Li H. A self-adaptive DRSN-GPReLU for bearing fault diagnosis under variable working conditions. Measurement Science and Technology. 2022;33(12):124005. doi: 10.1088/1361-6501/ac86e3

[29] Zhang Z, Chen L, Zhang C, Shi H, Li H. GMA-DRSNs: a novel fault diagnosis method with global multi-attention deep residual shrinkage networks. Measurement. 2022;196:111203. doi: 10.1016/j.measurement.2022.111203

